# Elucidating the Impact of Bacterial Composition and Diversity on Skin Homeostasis Using a Human 3D In Vitro Skin Model

**DOI:** 10.3390/microorganisms14081722

**Published:** 2026-08-05

**Authors:** Maya Sophie Kissner, Aline Rosin, Heike Sprenger, Giorgio Gonnella, Klaus Neuhaus, Amro Abbas, Hyun-Dong Chang, Tewes Tralau, Jannike Lea Krause, Tessa Höper, Lisa Lemoine

**Affiliations:** 1 Department of Pesticides Safety, German Federal Institute for Risk Assessment (BfR), 10589 Berlin, Germanyjannike.krause@bfr.bund.de (J.L.K.); hoeper@izw-berlin.de (T.H.); 2 Institute of Biotechnology, Technical University of Berlin, 13355 Berlin, Germany; 3 Department of Food Safety, German Federal Institute for Risk Assessment (BfR), 10589 Berlin, Germany; 4 Bioinformatics and AI Applications Unit, Institut Pasteur du Cambodge, Pasteur Network, Phnom Penh 120210, Cambodia; 5 Core Facility Microbiome, ZIEL—Institute for Food & Health, Technical University München, 85354 Freising, Germany; neuhaus@tum.de; 6 German Rheumatology Research Centre Berlin, A Leibniz Institute, 10117 Berlin, Germany; 7 German Federal Institute for Risk Assessment (BfR),10589 Berlin, Germany

**Keywords:** skin microbes, defined bacterial community, full-thickness skin model, bacterial skin colonization, host–microbiota interactions

## Abstract

The importance of the skin microbiome in human health has become increasingly evident. Despite considerable advancements, complex host–microbe interactions remain largely unexplored, primarily due to a paucity of models mimicking human skin and maintaining bacterial diversity in vitro. Therefore, we assessed the role of bacterial diversity for skin–microbiota co-cultivation in vitro by co-culturing reconstructed human full-thickness skin models with one of seven defined bacterial communities for seven days. The most diverse ten-strain community (BAP.10) comprised the three main skin phyla Bacillota, Actinomycetota, and Pseudomonadota. Lower diversity was obtained by the inoculation with two and one phyla combinations only. Inoculations with higher bacterial diversity were maintained at physiological bacterial counts and resulted in a higher functional similarity to human skin microbiomes, with BAP.10 showing the closest resemblance. In contrast, colonization with a single phylum led to either absence of viable bacteria or overgrowth of a single species. Overgrowth was associated with impaired skin integrity and cytotoxicity. Our data demonstrate the importance of bacterial diversity and the presence of certain genera for human-relevant skin co-culture models, which can be used for future in vitro studies elucidating skin–microbiome interactions.

## 1. Introduction

The human skin provides a physical barrier between the body and the environment, functioning as a primary defense against environmental noxae [1]. It harbors numerous microorganisms, including bacteria, fungi, viruses, and mites, collectively referred to as the skin microbiome [2]. Among these microorganisms, bacteria predominate in numbers. The main phyla present on human skin are Bacillota, Actinomycetota, and Pseudomonadota, and, with a lower abundance, Bacteroidota [3]. Skin commensals play a crucial role in cutaneous immune functions, contribute to pathogen defense [4,5,6], and regulate the formation and maintenance of the skin barrier [7,8]. Hereby, a diverse and stable skin microbiome is essential for its beneficial functions, as imbalances in its structure and function have been associated with skin disorders, such as atopic dermatitis and psoriasis [9].

The complex host–microbe interactions in healthy skin remain largely unexplored, primarily due to obstacles in mimicking human skin and maintaining bacterial diversity during co-cultivation in vitro. One approach is the co-cultivation at an air–liquid interface to create an in vivo-like environment for longer cultivation periods [10]. This approach was applied in previous studies, which focused on microbial pathogenesis and colonized organotypic skin with pathogens such as *Staphylococcus aureus* [11,12,13,14]. Other studies investigated the impact of selected commensals on skin cells and have found that microbe–host interactions affect tissue repair, cutaneous tolerance, and immune responses [15,16,17,18,19].

Understanding the impact of individual microbes on the host is valuable, yet a healthy skin generally depends on a diverse and balanced microbiome. In this regard, the utilization of defined microbial communities ensures a controllable and reproducible bacterial colonization that can be adapted to the respective research questions [20]. For murine skin colonization, defined skin microbiota from human donors have been established [21,22]. While these complex models allow the study of host–microbiota interactions in vivo, translatability issues remain a concern [23]. Consequently, researchers have employed human skin models inoculated with defined skin communities to enhance physiological relevance [24,25]. Although microbial diversity was not assessed experimentally at the end of the cultivation period, Loomis et al. [24] concluded that, compared to individual microbes, a diverse community more effectively influenced the expression of genes that affect key cell cycle processes, increased epidermal thickness, and enhanced cell proliferation in a full-thickness skin model. However, it remains uncertain whether the observed effects were attributable to the presence of the bacterial community as a whole, a single dominant species, or the absence of viable bacteria.

To study microbiome–skin interactions, co-culture models, which maintain bacterial diversity over several days and show functional compliance to the human skin microbiome without harming the skin tissue are required. To address this point, we analyzed microbiome–skin co-cultures colonized with defined bacterial communities of varying diversity on full-thickness skin after seven days with a focus on both, the microbiome and the skin. The defined bacterial communities comprised up to ten bacterial skin isolates from the three main skin phyla, Bacillota (B.4, four strains), Actinomycetota (A.4, four strains), and Pseudomonadota (P.2, two strains). To generate defined communities of different complexity with a comprehensible approach to investigate how bacterial diversity and the presence of certain genera may impact skin colonization in vitro, we divided the most diverse community into their phyla. We systematically evaluated the most diverse ten-strain BAP.10 community against combinations with a lower complexity of two phyla (BA.8, BP.6, and AP.6) or one phylum (B.4, A.4, and P.2) in terms of skin colonization, cutaneous processes, and functional compliance to the human skin microbiome.

Our findings underscore the relevance of bacterial diversity to providing in vivo-like metabolic functions. The most diverse BAP.10 community mimicked the functional profile of human skin microbiomes best and sustained greater diversity alongside physiological bacterial counts after co-cultivation, thereby preventing cytotoxicity and inflammation in the co-culture skin model. In contrast, for example, the one-phylum community B.4 was unable to colonize the skin models, potentially due to functional dependence on other species. Our results will be of value for future studies on skin–microbiome interactions in vitro.

## 2. Materials and Methods

### 2.1. Chemicals

All chemicals were obtained at the highest purity available from Sigma-Aldrich (Taufkirchen, Germany), unless otherwise specified.

### 2.2. Microbiological Methods

#### 2.2.1. Characterization and In Vitro Cultivation of Bacterial Skin Isolates

All ten bacterial isolates were derived from a previous study in which bacteria were isolated from human forearm skin samples and identified through whole-genome sequencing [26]. The choice of bacterial isolates was based on experimental requirements of the co-cultivation and practicalities of laboratory handling, i.e., aerobic co-cultivation with full-thickness skin models. The selected skin isolates and the respective community compositions are listed in Table 1.

The experiments were conducted prior to the reclassification of *Brevibacterium frigoritolerans* [27]; thus, *B. frigoritolerans*, now *Peribacillus simplex,* was part of the Actinomycetota communities in the present study.

Bacterial isolates were further characterized by analyzing their growth kinetics in duplicates of at least three biological replicates (Table S1). Therefore, liquid LB medium was inoculated with cryopreserved pure cultures and incubated at 32 °C with 200 rpm shaking overnight. The next day, cultures were transferred to fresh liquid LB medium and cultivated at 32 °C and 200 rpm. Bacterial growth was measured hourly using optical density (OD_600_ nm, FoodALYT/Omnilab, Bremen, Germany) and cell numbers were determined by counting colony forming units (CFUs). Therefore, bacterial suspensions were serially diluted, plated on LB agar plates and incubated at 32 °C. The doubling time (t_D_) was calculated based on the linear regression between CFU/mL and OD_600_ during the exponential growth phase.

For skin inoculation, LB cultures of all ten isolates were subjected to OD_600_ measurements and combined at the end of their exponential growth phase in equal cell numbers (Table 1) according to the predetermined relationship between OD_600_ and CFU/mL. Bacteria were washed twice with sterile DPBS (MatTek, Bratislava, Slovakia) and resuspended in a defined volume of DPBS.

#### 2.2.2. Bacterial Quantification

The quantification of bacterial cells used for skin model inoculation as well as the number of viable bacteria present at the end of the co-culture were determined using CFU counting and a cell counter (CASY TTC Cell Counter, TTC2LA2075, Roche Innovatis AG, Bielefeld, Germany). To ensure the absence of potential contaminants and their effects on the models, these procedures were also performed on the aseptic models. For bacterial quantification, all bacterial cultures were quantified in triplicates using serial dilutions (up to 1:10^8^) in DPBS, whereas bacterial communities collected on the harvest day were quantified using a single serial dilution per co-culture model.

Community performance was scored based on bacterial counts: desirable (3), within the physiological range (10^4^–10^8^ cell/1 cm^2^ skin); moderate (2), within the physiological range with minor outliers; and undesirable (1), outside the physiological range.

### 2.3. Co-Cultivation of Skin Models with Bacterial Communities

Commercial full-thickness 3D skin models (1 cm^2^, EpiDermFT™, EFT-400-ABF, MatTek, Bratislava, Slovakia) that consist of human epidermal keratinocytes and human dermal fibroblasts on cell culture inserts were utilized. These skin models have been validated by the European Centre for the Validation of Alternative Methods (ECVAM) and comply with test guidelines approved by the Organisation for Economic Co-operation and Development (OECD) [28]. The air–liquid-interface cultivation was conducted in six-well plates (Techno Plastics Products AG, Trasadingen, Switzerland) with 2.5 mL of antibiotic-free medium (EFT-400-MM-ABF-250, MatTek, Bratislava, Slovakia) at 5% CO_2_ and 37 °C with daily medium exchanges starting the day after delivery. Two days after delivery, skin models were inoculated with one of the seven bacterial communities.

**Figure 1:**
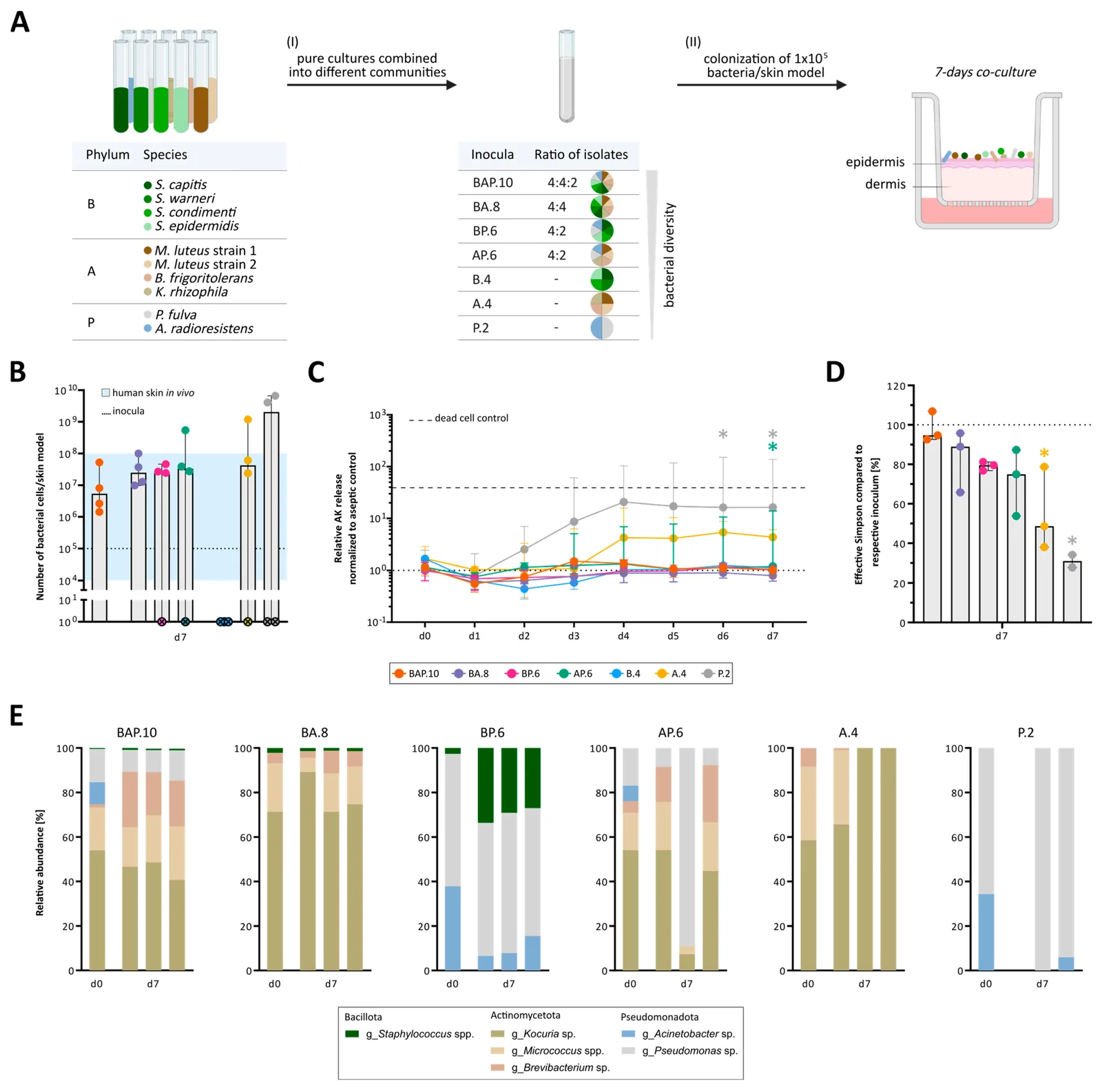
Inoculation and colonization of human skin models with bacterial communities of varying complexity. (**A**) Experimental setup: inoculation of skin models with bacterial communities. (**I**) Isolated bacteria were combined according to the respective phyla combinations with equal cell number ratios. (**II**) 1 × 10^5^ bacterial cells/skin model were applied on the apical side of the skin models and cultivated for seven days. (**B**) Number of viable bacterial cells isolated from co-culture skin models on day 7. Data are plotted as medians with 95% confidence interval (CI, n = 4). Circles with a cross refer to ‘no viable bacteria observed’. The dotted line indicates the average inoculum cell number and the blue background highlights the bacterial cell number range on human skin in vivo. (**C**) AK levels relative to the aseptic co-culture models over 7 days of cultivation as a measure for cytotoxicity. Data are presented as median with 95% CI (n = 4). The dashed line indicates the average AK release of the dead cell control. Differences between the single or two phyla co-cultures and the BAP.10 co-culture were analyzed using two-way ANOVA, followed by Dunnett’s multiple comparisons test and adjusted *p*-values are reported (* adj. *p* < 0.05). (**D**) α-diversity on day 7 shown as percentage of Simpson effective value compared to the respective inoculum. These were analyzed using multiple paired *t*-tests, * *p* < 0.05. Data are plotted as medians with 95% CI (n = 3). (**E**) Relative abundances at genus level on day 0 and day 7. The relative abundances on day 0 are displayed as mean of three biological replicates. Abbreviations: B = Bacillota; A = Actinomycetota; P = Pseudomonadota. Created in BioRender https://BioRender.com/1w6icbv; accessed on 10 June 2026.

For skin inoculation, the ten different skin bacteria were assembled into seven bacterial communities of varying diversity (Table 1 and Figure 1A). The most diverse bacterial community BAP.10 comprised all ten bacterial isolates at equal cell numbers covering the three phyla while resembling approximate phyla ratios as reported in the literature [29,30], with four Bacillota (B, 40%), four Actinomycetota (A, 40%) and two Pseudomonadota (P, 20%) strains, respectively. For two and one phylum communities, BA.8, BP.6, AP.6, B.4, A.4, and P.2, all strains were mixed at equal cell numbers as shown in Figure 1A and Table 1. Skin models were colonized with the respective communities at a final total concentration of 1 × 10^5^ bacterial cells in 15 µL DPBS per skin model (1 cm^2^). This concentration aligns with the range of bacterial cell numbers observed on human skin in vivo [31,32,33]. Aseptic models were treated with the same volume of sterile DPBS as a control. All co-cultures were cultivated for seven days with daily medium exchange (*n* = 4).

At the end of cultivation, the apical side of the skin models was gently washed with 300 µL DPBS to harvest the bacteria for quantification and 16S rRNA sequencing. The epidermis and dermis were separated, shock-frozen in liquid nitrogen, and stored at −80 °C for transcriptome analysis. Skin models for tissue sections were transferred to embedding medium (Sakura Finetek, Torrance, CA, USA), rapidly frozen in liquid nitrogen and stored at −80 °C.

### 2.4. Bacterial Community Analysis

#### 2.4.1. Bacterial DNA Extraction and Quantification

DNA was isolated from all bacterial communities prior to inoculation and after co-cultivation, as well as from aseptic models and reagents to capture the background, which included sterile DPBS, water, and kit controls. DNA was isolated using the QIAamp UCP DNA Micro Kit (QIAGEN GmbH, Hilden, Germany) following the manufacturer’s instructions. Nucleic acid concentrations were quantified using a UV-Vis Spectrophotometer (NanoDrop-ND 1000, Peqlab, Erlangen, Germany) and Qubit (Invitrogen, Carlsbad, CA, USA).

#### 2.4.2. 16S rRNA Gene Amplicon Sequencing

16S rRNA gene sequencing was conducted using a two-step polymerase chain reaction (PCR) protocol as described by Reitmeier et al. [34]. Briefly, for amplicon library preparation, the V1-V3 regions of the 16S rRNA gene [35] were amplified from 12 ng/µL isolated sample DNA using the forward primer 27F-YM-ovh (5′-TCG TCG GCA GCG TCA GAT GTG TAT AAG AGA CAG **AGA GTT TGA TYM TGG CTC AG**-3′) and reverse primer 534R (5′-GTC TCG TGG GCT CGG AGA TGT GTA TAA GAG ACA G**AT TAC CGC GGC TG G**-3′) [36,37]. In bold, the actual part of the primer binding to the 16S rRNA-genes is shown; the overhangs are needed for the second, barcoding PCR. The first amplicons were prepared in a 30 µL PCR with the following ingredients: 6 µL Phusion Buffer (Thermo Fisher Scientific, Waltham, MA, USA), 0.6 µL 20 mmol dNTPs, 0.1875 µL 20 mM forward primer, 0.1875 µL 20 mM reverse primer, 0.15 µL Phusion High-Fidelity DNA Polymerase Hotstart (Thermo Fisher Scientific, Waltham, MA, USA), 2.25 µL dimethyl sulfoxide (DMSO), and 17.625 µL water for molecular biology. The PCR settings were 30 s for initial denaturation at 98 °C, and then 15 cycles with 5 s denaturation at 98 °C, 10 s annealing at 55 °C, and 10 s extension at 72 °C. Subsequently, a final extension of 2 min at 72 °C was added, after which the PCR amplicons were ready for the second PCR. The second PCR added sequencing-adapters and barcodes for sequencing. For the second PCR, barcoded primers were used, which contain an adapter for the Illumina sequencing (P5 and P7), a barcode of 8 nt shown in bold as N8 according to [38], and a primer part able to bind to the overhangs of the first primer, respectively, P5-BC-ovh (5′-AAT GAT ACG GCG ACC ACC GAG ATC TAC AC**N NNN NNN N**TC GTC GGC AGC GTC-3′) and P7-BC-ovh (5′-CAA GCA GAA GAC GGC ATA CGA GAT **NNN NNN NN**G TCT CGT GGG CTC GG-3′). For the second PCR, 10 µL Phusion HF Buffer, 1 µL 20 mmol dNTPs, 0.313 µL 20 mM forward primer, 0.313 µL 20 mM reverse primer, 0.2 µL Phusion High-Fidelity DNA Polymerase Hotstart, 1.5 µL DMSO, and 32.487 µL water for molecular biology were mixed. As the template, 2 µL of the first PCR were added. The second PCR was run using 30 s for initial denaturation at 98 °C, and then 10 cycles with 5 s denaturation at 98 °C, 10 s annealing at 55 °C, and 10 s extension at 72 °C. Subsequently, a final extension of 2 min at 72 °C was added, after which the PCR amplicons were ready for cleanup. PCR purification was performed with AGENCOURT AMPure XP Beads (Beckman Coulter, Krefeld, Germany) according to the manual. Final amplicons were pooled equimolarly to a concentration of 20 pM. This pool was denatured, diluted to 10 pM, and spiked with 10% phiX (Illumina PhiX Control v3, Illumina Inc., San Diego, CA, USA), according to the instruction of the manufacturer for the MiSeq Illumina sequencer (Illumina Inc., San Diego, CA, USA). The final library was sequenced on a MiSeq system using PE300 cartridges v3 with 2 × 275 cycles (Illumina Inc., San Diego, CA, USA), following the manufacturer’s instructions.

#### 2.4.3. Data Analysis of the 16S rRNA Gene Amplicon

Raw reads were trimmed 5 bp on either side [39] and further processed using the Integrated Microbial Next-generation sequencing 2 (IMNGS2.org) pipeline [40]. IMNGS2 performs the Taxonomy Informed Clustering (TIC) algorithm for enhanced taxonomic resolution, generating species-like operational taxonomic units (SOTUs) [41], which are denoised zOTUs clustered within each identified genus. For subsequent analysis, only SOTUs with a relative abundance ≥0.25% in at least one sample were retained [42]. In addition, SOTUs recognized as typical contaminants [43] and contaminants of reagent samples [44], were removed from all samples as this is highly relevant for low biomass samples. The annotation of the 16S amplicon sequences was performed using EzBioCloud’s 16S rRNA gene-based ID [45]. Rhea v1.1.8, implemented in the R environment v4.2.3, was utilized to evaluate α-diversity based on species richness, Shannon, and Simpson effective values [46]. β-diversity was analyzed by Bray–Curtis dissimilarity (n = 3).

Community performance was scored based on maintenance of bacterial diversity compared to their inoculum: desirable (3), α-diversity loss <20%; moderate (2), α-diversity loss <40%; and undesirable (1), α-diversity loss >40%.

### 2.5. Functional Profiling

#### 2.5.1. KEGG Ontology and Pathways Annotation

Kyoto Encyclopedia of Genes and Genomes (KEGG) ontology (KO) functional annotations were computed for each bacterial isolate using KofamScan v1.3.0 [47] sets, using HMMER v3.4 [48] profile searches. Carbohydrate-active enzyme (CAZy) potential was summarized from dbCAN [49] annotations per isolate, combining HMMER [48] and DIAMOND [50] searches, with CAZy family richness counted from parsed family identifiers and class distributions grouped by glycoside hydrolase (GH), glycosyltransferase (GT), polysaccharide lyase (PL), carbohydrate esterase (CE), auxiliary activity (AA), and carbohydrate-binding module (CBM) prefixes. Biosynthetic gene clusters were predicted per isolate with antiSMASH v8.0.4 [51], including the MITE database v1.3 [52]. Antimicrobial resistance (AMR) determinants were identified using RGI v6.0.5 against the CARD database v6.0.5 [53] and AMRFinderPlus v4.2.7 against the NCBI AMRFinderPlus database (2025-12-03.1) [54]. Virulence factor genes were identified using ABRicate v1.4.0 [55] against the VFDB core dataset [56].

For each bacterial community, these annotations were pooled and mapped to KEGG modules [57]. Thereby, we selected KEGG modules present in bacteria with >300 hits from the KEGG module database [58].

#### 2.5.2. Comparison with Selected Volar Forearm Metagenome-Assembled Genomes of the Skin Microbial Genome Collection

The functional potentials of each defined community in terms of the above-mentioned KEGG modules were compared with metagenome-assembled genomes (MAGs) from selected samples of the Skin Microbial Genome Collection (SMGC) [30]. Therefore, we selected SMGC samples exclusively from the volar forearm as follows. The list of non-redundant prokaryotic MAG sets and the list of co-assembly units were obtained from the SMGC website (‘prok_nonredundant.csv’ and ‘sample_metadata.txt’). Matching their information, we computed SRA run IDs or BioSample IDs (for Met IDs using the information from Saheb Kashaf et al. [30]) for each co-assembly unit, and obtained the body site location from NCBI SRA queries. We then selected only MAGs which were assembled from a co-assembly unit consisting of only volar forearm samples and no samples from other body sites, leading to a final list of 188 MAGs, which were used for our comparison. Analogously to what was done for our isolates, we computed KOs for the selected SMGC MAGs with KofamScan v1.3.0. Pairwise functional similarity between the seven defined communities and the pooled SMGC reference was quantified as the Jaccard similarity of pooled KO sets (intersection-over-union of KO sets). These KO sets were then mapped to the (skin) bacteria-relevant KEGG modules mentioned above. The resulting module count matrix, column-normalized to relative composition and z-score standardized, was decomposed by principal component analysis (PCA) to visualize the similarity of the seven communities to the pooled SMGC volar forearm reference. For the direct functional comparison, we calculated the completeness of those KEGG modules as a proxy for the ability of the communities/the SMGC to fulfill the subsequent functions for heatmap visualization. The heatmap was rendered with Matplotlib v3.11.0 [59] using Euclidean distance, with average-linkage hierarchical clustering, and optimal leaf ordering as computed by scipy v1.17.1 [60].

Community performance was scored based on functional similarity to the human skin microbiome: desirable (3), high similarity to the SMGC (Jaccard index >0.6); moderate (2), moderate similarity to the SMGC (Jaccard index between 0.5–0.6); and undesirable (1), low similarity to the SMGC (Jaccard index <0.5).

### 2.6. Transcriptome Analysis of Skin Tissues

#### 2.6.1. RNA Extraction and Sample Preparation for Microarray Gene Analysis

Total RNA was isolated from both epidermis and dermis of three biological replicates using a TRIzol-based protocol according to the manufacturer’s instructions [61]. At first, tissues were disrupted twice by bead beating at 25 Hz for 5 min in a TissueLyser II (Qiagen, Hilden, Germany) using TRIzol^TM^ (Invitrogen, Carlsbad, CA, USA). Final concentrations of the isolated RNA were measured using a NanoDrop-ND 1000 (Peqlab, Erlangen, Germany). RNA integrity (RIN) was determined using the Agilent RNA 6000 Nano Kit on an Agilent Bioanalyzer System (Agilent Technologies, Waldbronn, Germany).

A total of 500 ng of total RNA was used for microarray gene expression analysis using Human Clariom^TM^ S Assays (Applied Biosystems, Foster City, CA, USA) performed at ATLAS Biolabs (Berlin, Germany). The Human Clariom^TM^ S assay was used to enable robust, standardized analysis of approximately 20,000 human genes. This approach was chosen to characterize changes in global gene expression and associated biological signaling pathways induced by colonization with different bacterial communities. Labeling of the samples with terminal deoxynucleotidyl transferase (TdT) was carried out using a proprietary DNA labeling reagent at ATLAS Biolabs (Berlin, Germany).

#### 2.6.2. Data Processing for Microarray Analysis

Microarray data were analyzed and visualized within R v4.4.0 [62]. The R package arrayQualityMetrics v.3.60.0 was used to perform quality control of all microarray files [63]. Raw data (CEL files) were normalized and summarized using the robust multiarray average (RMA) algorithm from the R package oligo v1.68.2 [64]. Annotation of probes was performed with the vendor-provided array information and control probes were removed after background correction. For further analysis, genes with low expression were removed, such that 21,194 probes corresponding to 19,304 genes remained with an intensity > 4 in at least two samples. Subsequently, differential gene expression analysis was conducted on the normalized dataset using limma v3.60.2 in R [65]. The eBayes function was employed to fit linear models and calculate moderated t-statistics, followed by Benjamini–Hochberg correction to exclude false positives. Genes were considered significantly differentially expressed when they met the following criteria: an adjusted *p*-value < 0.05 and a log_2_FC > 0.5 or <−0.5. PCA was applied to batch-corrected, pareto-scaled, and centered data using the R package pcaMethods v1.96.0 [66]. Because the samples were sequenced in multiple sets, resulting in technical artifacts, the batch effect was corrected using the removeBatchEffect function from the limma package before performing PCA. The global rotation test (fry) within the limma package was employed to evaluate the global epidermal transcriptome variations between A.4 co-cultures and other co-cultures. The mixed-direction *p*-value (p.mixed) was utilized to examine overall differences in expression profiles, regardless of the direction of changes in individual genes. Gene set enrichment analysis (GSEA) was performed using the gseGO function from the clusterProfiler package v4.12.0 [67] to identify enriched gene ontology (GO) terms for biological processes [68]. GO terms with an adjusted *p*-value < 0.05 were considered significant and normalized enrichment scores (NES) of top-ranked GO terms were visualized in treeplots from the R package enrichplot v1.26.6 [69]. Heatmaps were generated with pheatmap v1.0.12 in R.

### 2.7. Quantification of Released Compounds

To assess the overall viability of the co-culture models as a whole, cytotoxicity was monitored daily by measuring the levels of adenylate kinase (AK) released into the basolateral medium in technical duplicates using the ToxiLight^®^ BioAssay Kit (Lonza, Basel, Switzerland) following the manufacturer’s instructions. Moreover, the secretion of fibroblast growth factor (FGF) into the medium was quantified in technical duplicates across three biological replicates utilizing the Human FGF basic/FGF2/bFGF ELISA Kit (R&D Systems, Minneapolis, MN, USA) according to the manufacturer’s instructions.

Community performance was scored based on cytotoxicity: desirable (3), no cytotoxicity and mean AK levels were <5% compared to the dead cell control; moderate (2), no cytotoxicity with mean moderate AK levels between 5 and 30% compared to the dead cell control; and undesirable (1), high AK levels with mean AK levels >30% compared to the dead cell control.

### 2.8. Tissue Staining

Cryopreserved tissues were sectioned using the cryotome Cryostat Microm HM550 (Thermo Fisher Scientific, Waltham, MA, USA) and mounted on Superfrost slides (Thermo Fisher Scientific, Waltham, MA, USA). For hematoxylin and eosin (HE) staining, the H&E Staining Kit (Abcam, Cambridge, UK) was used according to the manufacturer’s instructions. The stained slides were subsequently visualized using the BZ-X microscope (KEYENCE, Neu-Isenburg, Germany). Images were used to quantify epidermal thickness. Accordingly, ten distinct and randomly selected intact epidermal regions were measured across three different skin models for each group using ImageJ v1.53.

For immunostaining, tissue sections were fixed in 100% ice-cold methanol, then washed and rehydrated for 5 min using DPBS (Pan-Biotech, Aidenbach, Germany). Tissues were permeabilized in 0.5% TritonX-100 (*v*/*v*) dissolved in DPBS for 9 min, then rinsed twice with DPBS and blocked with 0.2% Tween (*v*/*v*) and 3% bovine serum albumin (BSA) (*w*/*v*) in DPBS for 40 min. This was followed by incubation with one of the below listed primary antibodies in blocking solution for 2 h, washing with DPBS and incubation with an adequate secondary antibody in blocking solution for 50 min. As primary antibodies, E-cadherin was diluted 1:30 (33–4000; Thermo Fisher Scientific, Waltham, MA, USA) and Cytokeratin 10 1:50 (18343-1-AP; Proteintech, Manchester, UK) in blocking solution. The following secondary antibodies were used: Alexa fluor 488 Goat anti-Rabbit IgG (H + L) diluted 1:200 (A-11008, Thermo Fisher Scientific, Waltham, MA, USA) and Alexa fluor 488 Goat anti-Mouse IgG (H + L) diluted 1:400 (A-11001, Thermo Scientific, Waltham, MA, USA). Following antibody binding, the tissues were washed thrice with DPBS containing 0.2% Tween, followed by a final rinse in DPBS. Finally, the sections were counterstained with 1 µg/mL Hoechst in DPBS for 10 min and mounted using FluorSave Reagent (Sigma-Aldrich, Taufkirchen, Germany). The stained slides were analyzed using a LSM700 confocal microscope (Carl Zeiss, Oberkochen, Germany). The “Tile Scan” function in the ZEN 2012 black edition software (Carl Zeiss, Oberkochen, Germany) was used to employ a 2 × 2 tile configuration.

Community performance was scored based on skin barrier integrity: desirable (3), increased epidermal thickness and skin barrier marker expression compared to aseptic control; moderate (2), comparable or slightly reduced levels; and undesirable (1), impaired or reduced epidermal thickness and skin barrier marker expression.

### 2.9. Visualization and Statistical Analysis

Data visualization and statistical analyses were conducted using R v4.2.3 or GraphPad Prism v9.3.1 (Graph Pad, La Jolla, CA, USA) with significance levels of * *p* < 0.05, ** *p* < 0.01, *** *p* < 0.001, and **** *p* < 0.0001, respectively. Details of each statistical analysis are provided in the respective method descriptions and figure legends.

To evaluate the overall performance of the bacterial communities in colonizing the skin models, the communities were assigned a score for each conducted analysis ranging from one to three, with 3 = desirable, 2 = moderate, and 1 = undesirable outcomes, with respect to maintaining a healthy in vivo-like human skin condition. The individual scores, along with their means, were visualized in a heatmap.

The graphical illustration of the experimental setup was created in BioRender.

## 3. Results

In this study, we examined how bacterial communities with differing diversity colonize human full-thickness skin models (Table 1, Figure 1A) and assessed their suitability to study interactions between host and skin bacteria in vitro.

### 3.1. Higher Bacterial Diversity Promotes Stable Skin Colonization

The initial number of viable bacteria was 1 × 10^5^ bacterial cells per skin model (Figure 1B, dotted line). Following seven days of co-cultivation, the BAP.10 and BA.8 co-cultures displayed bacterial cell counts that ranged from 10^6^ to 10^8^ cells per skin model, which is comparable to the bacterial load on human skin in vivo (Figure 1B, blue background). While for these communities, the cell counts were homogenous across all replicates, this was not the case for skin models inoculated with BP.6, AP.6, A.4, and P.2 communities. In these co-cultures, viability differed with some biological replicates not containing viable bacteria and others harboring high bacterial numbers on the day of harvest. Skin models inoculated with communities of only one phylum exhibited the most pronounced differences between replicates. While no viable bacteria were observed for co-cultures inoculated with the B.4 community, strong bacterial growth was observed for co-cultures inoculated with the P.2 community, exceeding 10^9^ bacterial cells per skin model. Aseptic models did not show bacterial growth. B.4 co-cultures, inoculated solely with Bacillota species, were excluded from further analyses due to the absence of viable bacteria at the end of cultivation.

The viability of the co-cultures, which comprised microbiota and skin tissue cells, was monitored daily by the release of AK into the culture medium as an indicator of cytotoxicity (Figure 1C). Lysed skin models were used as a dead cell control indicative of high cytotoxicity (Figure 1C, dashed line). The BAP.10, BA.8, BP.6, and B.4 co-cultures did not exhibit any signs of cytotoxicity throughout the cultivation period. Compared to BAP.10 co-cultures, the AP.6 and A.4 co-cultures showed a significantly higher AK release on day 7 and the P.2 co-cultures from day 6 onwards, reaching levels comparable to the dead cell control.

Next, 16S rRNA gene amplicon sequencing was performed to examine α- and β-diversities as well as the taxonomy of the bacteria (Figure 1D,E and Figure S1). Given the known composition of the bacterial communities, the effective Simpson value was selected to assess α-diversity, as it emphasizes abundant species and is less sensitive to rare species [70]. The BAP.10 and BA.8 co-cultures did not exhibit relevant diversity reduction over the cultivation period with reductions to 95% and 89% effective Simpson value, respectively (Figure 1D). In contrast, reduction of effective Simpson values was more pronounced for the remaining bacterial communities, with a significant decrease in A.4 and P.2 co-cultures.

The taxonomic profiles of BAP.10, BA.8, and BP.6 communities demonstrated a reproducible bacterial composition maintaining most genera from the inoculum on day 7 (Figure 1E; Table S3). The BAP.10 community on day 7 exhibited a relative increase in *Brevibacterium* sp. and a decrease in *Acinetobacter* sp. compared to the inoculum. The BP.6 co-culture showed a notable increase in *Staphylococcus* spp. and a decrease in *Acinetobacter* sp. compared to the inoculum. Similar to the varying apical bacteria numbers, the composition of the AP.6, A.4, and P.2 co-cultures exhibited greater variability across replicates. In two co-cultures of AP.6, a similar composition was observed, whereas one co-culture demonstrated a relative predominance of *Pseudomonas* sp. and an absence of both *Brevibacterium* sp. and *Acinetobacter* sp. This AP.6 co-culture also exhibited an elevated bacterial growth (Figure 1B). Notably, only one A.4 co-culture retained all initial taxa at the end of cultivation. In the viable P.2 co-cultures, *Pseudomonas* sp. emerged as the predominant genus. Notably, the relative abundance of *Acinetobacter* sp. decreased in all *Acinetobacter*-containing co-cultures compared to the inocula. This was especially pronounced in the presence of bacteria from Actinomycetota in the inoculum and the P.2 co-culture. Moreover, solely BP.6 co-cultures showed an increase in the relative abundance of *Staphylococcus* spp., whereas *Staphylococcus* spp. were not dominant in any of the other co-cultures that contained Actinomycetota.

Overall, low diversity resulted in an overgrowth of a single species or lack of colonization ability, a decrease in α-diversity, and elevated cytotoxicity induction at the end of the co-cultivation. These findings suggest that a balanced skin colonization in terms of bacterial cell number, community composition, and co-culture viability depends on high microbial diversity.

### 3.2. Functional Profiling of Bacterial Communities Compared to the Skin Microbiome Genome Collection (SMGC)

Although our personal microbiome is as unique as a fingerprint, a healthy microbiome harbors a high species diversity to exert specific metabolic functions [71,72]. As host–microbiome interactions are based on microbial metabolism, we compared the functional potential of all seven microbial communities, computed from the whole-genome sequences of all individual species, to realistic skin microbiomes from the SMGC.

Based on gene-level annotation, the four Bacillota, i.e., *Staphylococcus* spp., encoded similar numbers of KO terms (1235–1332) and CAZyme families (18–21), while species differences among the Actinomycetota and Pseudomonadota were apparent (Figure S2). *B. frigoritolerans* (Actinomycetota) and *P. fulva* (Pseudomonadota) by far provided the highest number of different KO terms (2146 and 2214, respectively, Figure S2A) and coded for the highest number of CAZyme families (47 and 37 CAZyme families, respectively, Figure S2B). On average, Pseudomonadota encoded the highest number of functional genes of all phyla.

**Figure 2:**
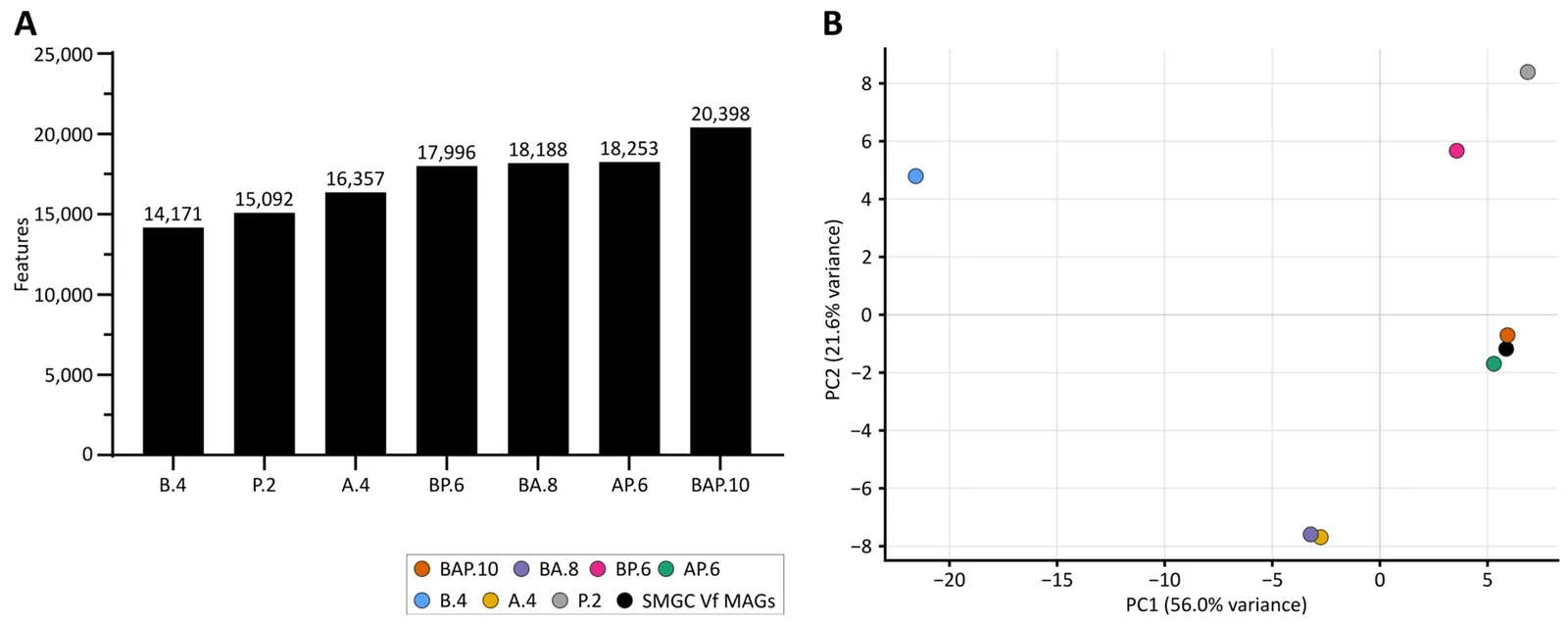
Genome-based functional potential analysis. (**A**) Number of distinct functional features (KO terms, CAZyme families, biosynthetic gene clusters, AMR/virulence gene hits) pooled across isolates of the seven bacterial communities. (**B**) PCA of bacteria-relevant KEGG module composition, comparing the seven communities to the pooled SMGC volar forearm reference. Abbreviations: B = Bacillota; A = Actinomycetota; P = Pseudomonadota; SMGC Vf MAGs = Skin Microbial Genome Collection metagenome-assembled genomes.

Next, the functional potential of the bacterial communities was computed based on the number of distinct functional annotation features (KO terms, CAZyme families, biosynthetic gene clusters, and AMR/virulence gene hits), encoded by their members. The single phylum community B.4 possessed the lowest number of functional features (14,171) followed by P.2 (15,092) and A.4 (16,357) (Figure 2A). Among the two phyla communities, the number of functional features were rather similar, with the community BP.6 showing the lowest functional potential and AP.6 the highest. Unsurprisingly, the most diverse community that included all three phyla/ten strains, BAP.10, proved the highest functional potential. The communities’ functional potential was also mirrored in the similarity of the seven communities to the volar forearm microbiomes extracted from the SMGC (Figure 2B, Figures S3 and S4).

The BAP.10 as well as the AP.6 community demonstrated high similarity to the SMGC. Despite the comparable number of KEGG functional categories provided by the other two phyla communities, the BP.6 and BA.8 communities were distinct from the SMGC. Although lacking the four Bacillota species, the A.4 community showed the same functional similarity to the SMGC as the BA.8 community suggesting a superior functional relevance of the Actinomycetota species for skin-relevant bacterial functions, especially when compared to B.4. The B.4 community was functionally most distinct to the SMGC and the other two- and single-phyla communities (Figure 2B, Figures S3 and S4). When comparing the completeness of annotated KEGG modules (Figure S4), the data indicate that B.4 has limited capacity in ‘aromatic amino acids metabolism’, ‘ATP synthesis’, ‘nucleotide sugar metabolism’, and ‘lipid metabolism’. This, together with the inability to colonize the skin models alone, suggests a metabolic dependence of the B.4 community, and with that, an inability to provide the functions of real skin microbiota, and assumingly, to successfully colonize our skin models in vitro. In contrast, and although different from the SMGC, the single-phylum community P.2, comprising *P. fulva* and *A. radioresistens*, had a low number of incomplete KEGG modules (Figure S4). In conjunction with the B.4 community, the BP.6 community showed a higher similarity to the SMGC, despite the lack of Actinomycetota. However, the functional relevance of Actinomycetota was mirrored in the high similarity of the AP.6 to the SMGC, despite the A.4 and P.2 community both being functionally distinct. The BAP.10 together with the AP.6 community showed high functional similarity to the SMGC, undermining the subordinate role of Bacillota for the functional potential of the BAP.10 community. However, compared to the AP.6 community, the BAP.10 showed slightly higher KEGG module completeness (Figure S4).

In summary, we hypothesized that the presence of certain bacteria of Actinomycetota are essential to provide key functions of the skin microbiome. Further, our data demonstrate that a higher diversity including Actinomycetota favors the full coverage. In contrast, bacterial isolates of Bacillota alone were insufficient to replicate the functions of human skin microbiomes.

### 3.3. Skin Colonization with Bacterial Communities of Differing Diversity Results in Distinct Tissue Gene Expression

To gain insights into the effects of bacterial skin colonization on gene expression relevant for skin microbiome interaction, the transcriptomic responses of the epidermis and dermis of colonized and aseptic tissues were analyzed using Human Clariom^TM^ S gene arrays, which examine 20,000 annotated human genes. Aseptic tissues were used as a reference, while skin tissues inoculated with the B.4 or P.2 community were excluded from this analysis due to the absence of viable bacteria in all or half of the co-cultures.

**Figure 3:**
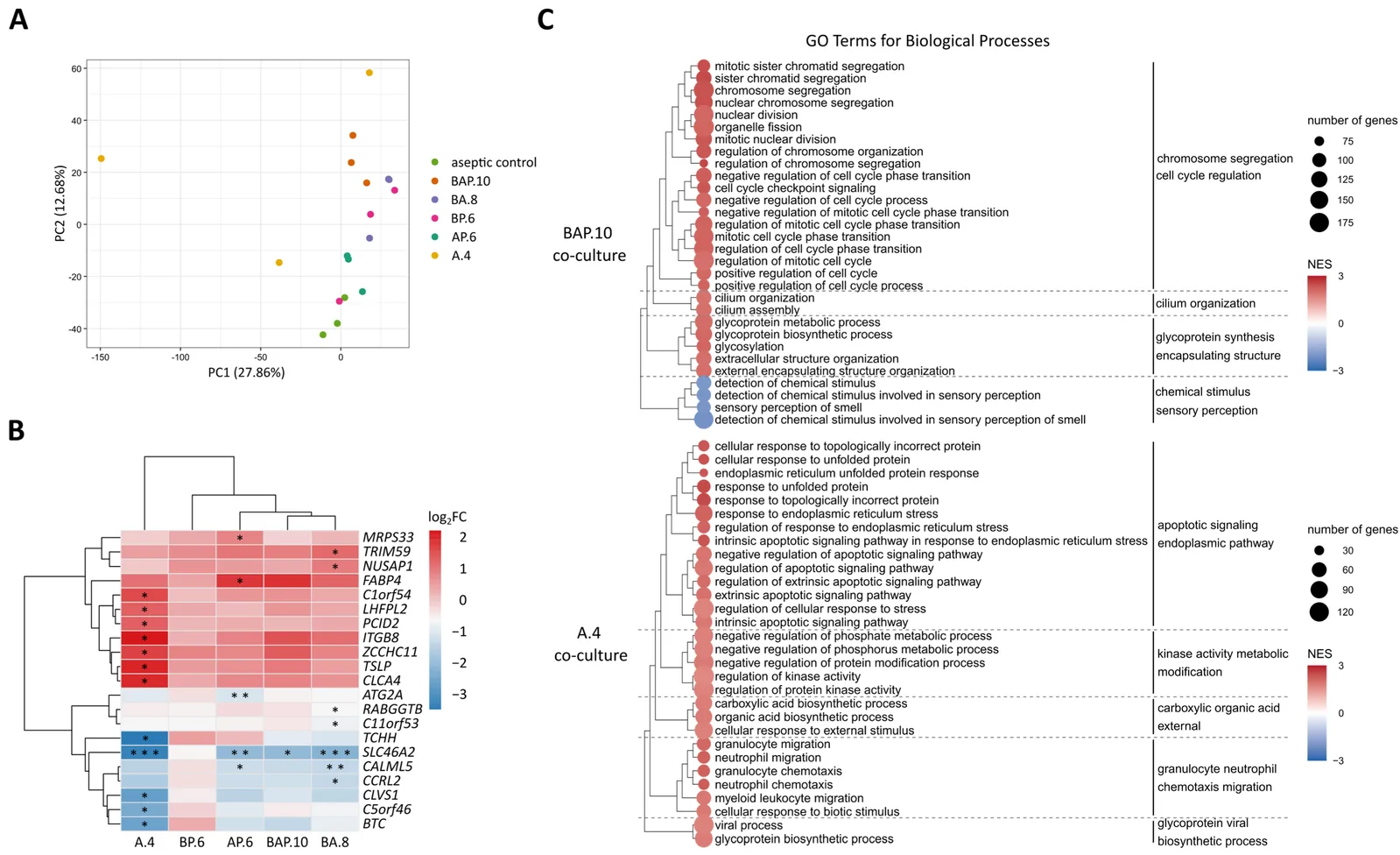
Epidermal transcriptomic profiles of colonized and aseptic tissues. (**A**) Similarity of epidermal transcriptomic profiles on day 7 was analyzed using PCA. (**B**) Log_2_FC of all differentially expressed genes in the epidermis of colonized tissues compared to aseptic tissue was visualized in a heatmap with cluster dendrograms for both genes and microbial communities. (**C**) Treeplots of gene set enrichment analysis (GSEA) identifying enriched gene ontology (GO) terms for biological processes in the epidermis of BAP.10 and A.4 co-cultures normalized to aseptic tissue on day 7 (n = 3). The dot size represents the number of genes per functional group and the color code refers to the normalized enrichment score (NES). Significance levels are presented as adjusted *p*-value with * adj. *p* < 0.05, ** adj. *p* < 0.01, and *** adj. *p* < 0.001. Abbreviations: B = Bacillota; A = Actinomycetota; P = Pseudomonadota.

PCA of gene expression profiles revealed pronounced clustering according to the different colonization conditions in the dermis (Figure S5A), which was not as pronounced in the epidermis (Figure 3A). Epidermal tissue colonized exclusively by bacterial isolates of Actinomycetota showed significant divergence in replicate transcriptomic profiles compared to the other co-cultures and exhibited the highest number of differentially expressed genes after seven days (Figure 3B and Figure S5A,B; Table S2, p.mixed = 0.042). Genes associated with antimicrobial response were upregulated in these tissues compared to the other co-cultures (Figure S6A). Tissues colonized by bacterial isolates of Actinomycetota alongside other phyla also exhibited elevated numbers of differentially expressed genes, potentially due to the presence of Actinomycetota species (Figure 3B and Figure S5B), with BAP.10- and BA.8-colonized epidermal tissues exhibiting a low number of differentially expressed genes compared to the aseptic tissue (Figure 3B). No significant differences were observed in the dermal tissue (Figure 3B and Figure S5B). Apart from the A.4-colonized tissue, only AP.6-colonized tissue showed a consecutive gene induction in the dermis (Figure S5B).

Skin tissues colonized by A.4 showed a significantly distinct transcriptomic response compared to the other co-cultures (Table S2), accompanied by low species diversity and elevated cytotoxicity. Hence, they were selected as a reference for a non-physiological condition and were subjected to a more detailed comparison with tissue colonized by the most diverse community (BAP.10). GSEA, a pathway enrichment analysis, was conducted to identify differential GO terms for biological processes upon co-cultivation with the two respective communities (Figure 3C and Figure S5C). Tissue colonized by BAP.10 showed upregulation of GO terms associated with ‘chromosome segregation and cell cycle regulation’ and ‘glycoprotein synthesis and encapsulating structure’ in the epidermis as well as ‘development and cell differentiation’ and ‘nuclear transport and ribosome biogenesis’ in the dermis. The upregulation of these biological processes suggests tissues homeostasis and renewal, indicating that colonization by the BAP.10 community is well tolerated by the skin model. Downregulation of genes involved in ‘chemical stimulus and sensory perception’ was observed in both, epidermis and dermis. In contrast, the GSEA in A.4-colonized tissues suggested a non-physiological condition in these co-culture models, as pathways associated with ‘apoptotic signaling’ and ‘kinase activity and metabolic modification’ were activated in the epidermis, while ‘cellular macroautophagy’ and ‘intrinsic negative apoptotic modification’ was observed in the dermis. Furthermore, the induction of the GO terms related to ‘granulocyte or neutrophil chemotaxis and migration’ in the epidermis potentially indicate the presence of infection or inflammation in the A.4 co-cultures.

In summary, the tissues colonized with the A.4 community induced distinct transcriptomic response, accompanied by consecutive induction of genes that may correspond to a non-physiological condition. This was not observed in tissues colonized by combinations of two or three phyla communities.

### 3.4. Higher Bacterial Diversity May Strengthen the Skin Barrier

**Figure 4:**
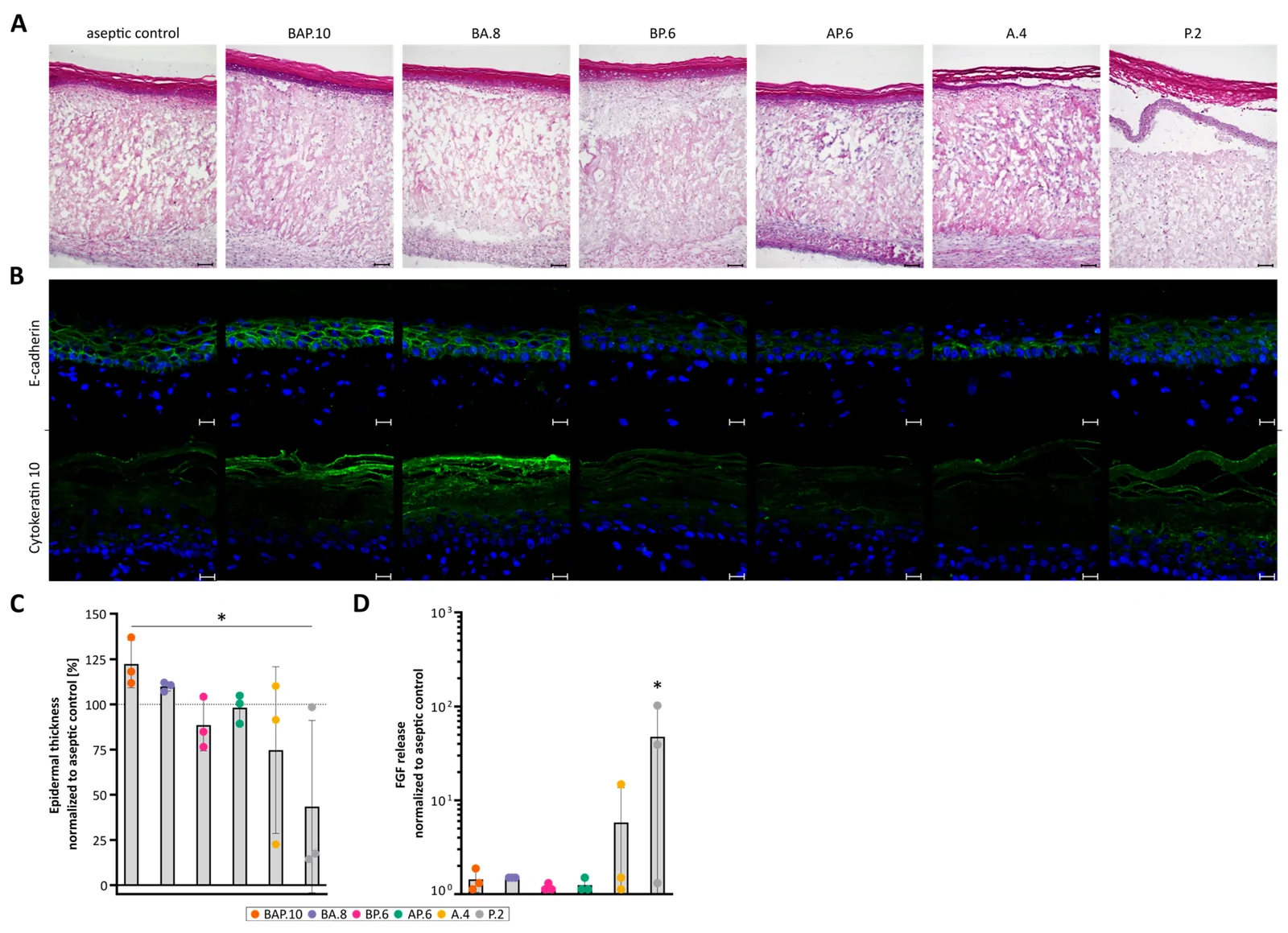
Barrier integrity of colonized and aseptic models. Representative images of inoculated and aseptic models on day 7 of cultivation. (**A**) HE staining; scale bars indicate 100 µm. (**B**) Immunostaining of E-cadherin and Cytokeratin 10 (green), and the cell nuclei with Hoechst (blue); scale bars indicate 20 µm. (**C**) Epidermal thickness was measured across ten different regions of the HE staining (n = 3). Differences between co-cultures were analyzed using one-way ANOVA, followed by Tukey’s multiple comparisons test. Adjusted *p*-values are reported (* adj. *p* < 0.05). (**D**) Relative quantification of released fibroblast growth factor (FGF) into the supernatant of colonized models normalized to aseptic control on day 7 based on ELISA measurements (n = 3). Data shown as means with ± SD. Differences between the aseptic control and the inoculated co-culture were analyzed using one-way ANOVA, followed by Dunnett’s multiple comparisons test. Adjusted *p*-values are reported (* adj. *p* < 0.05). Abbreviations: B = Bacillota; A = Actinomycetota; P = Pseudomonadota.

The impact of bacterial colonization on the skin barrier was characterized and compared to aseptic models by HE staining to visualize relevant tissue structures (Figure 4A and Figure S7). Colonization by either the A.4 or P.2 community resulted in impaired tissue structures with a detached *stratum corneum*. In contrast, skin models colonized by BAP.10 or by a combination of two phyla maintained structural integrity, exhibiting a notably denser epidermis and *stratum corneum* in the case of BAP.10 co-cultures. These observations were corroborated by quantitative analysis of the epidermal thickness in the HE-stained sections (Figure 4C, Figures S7 and S8A). The BAP.10 co-cultures, and to a lesser extent the BA.8 co-cultures, demonstrated a marginally increased epidermal thickness compared to aseptic models. Conversely, the P.2 co-cultures exhibited a notable reduction in epidermal thickness up to a significant level when compared to the BAP.10 co-cultures. These findings suggest that increased microbial diversity preserves the skin structure during co-cultivation.

We next analyzed the expression of the differentiation marker Cytokeratin 10 [73] and the protein E-cadherin, which is essential for the formation of tight-junctions [74], to further investigate effects on the epidermal barrier. Immunostaining against both markers in BAP.10 and BA.8 co-cultures visualized their elevated expression, while BP.6 and AP.6 co-cultures exhibited the weakest signal of all (Figure 4B). These findings were in line with the transcriptional upregulation of epidermal barrier marker genes associated with skin barrier maintenance and function in BAP.10 co-cultures (Figure S6B).

Furthermore, we quantified FGF, which is involved in tissue regeneration and repair [75] and serves as a marker to investigate possible cytotoxic effects. Elevated FGF levels were observed in A.4 and P.2 co-cultures at the end of the cultivation period, with a significant increase for the P.2 co-culture compared to the aseptic models (Figure 4D and Figure S8B). Interestingly, the reduced epidermal thickness in A.4 and P.2 co-cultures was mirrored by an increase in FGF levels. The other co-cultures exhibited FGF levels comparable to those observed in aseptic models.

Overall, the structural integrity was maintained and the skin barrier function was enhanced in skin models colonized by the BAP.10 or BA.8 community. In contrast, colonization by the other communities did not enhance the skin barrier or, in the case of colonization by A.4 or P.2 communities, led to compromised skin integrity.

### 3.5. Summary

**Figure 5:**
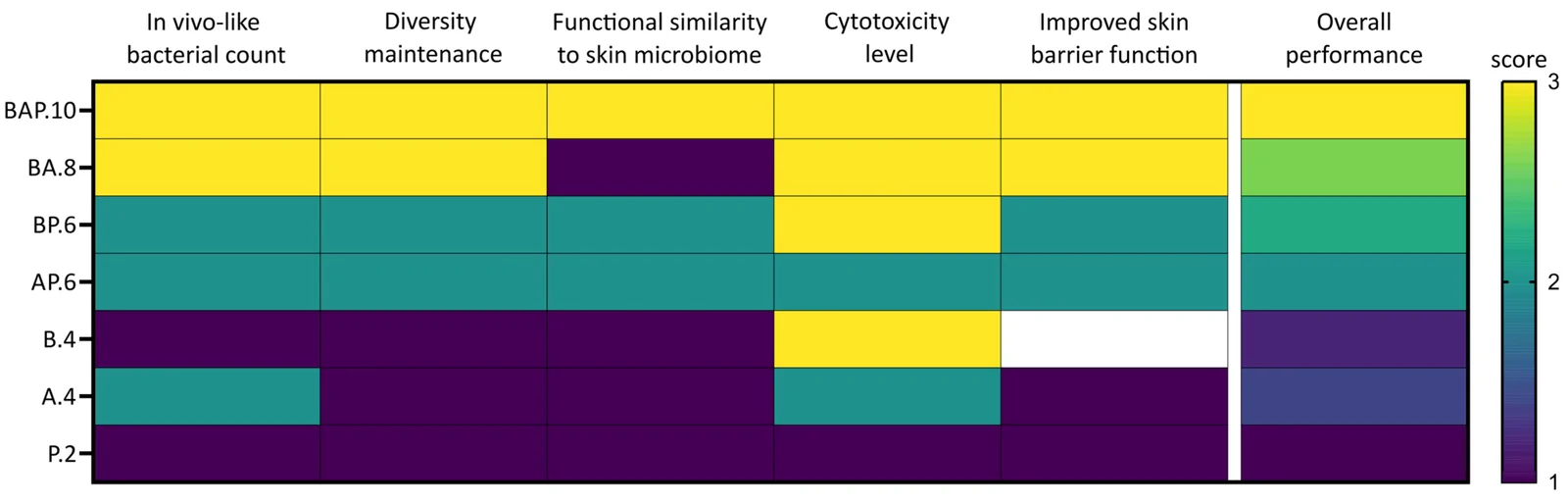
Overall performance of all bacterial communities during co-cultivation with reconstructed human skin in vitro summarized in a heatmap. The overall performance is displayed as the average color of the various analyses. The color code score is as follows: 3 = desirable, 2 = moderate, and 1 = undesirable findings. White: excluded from analyses due to absence of viable bacteria. Abbreviations: B = Bacillota; A = Actinomycetota; P = Pseudomonadota.

The main findings of this study are summarized in Figure 5, along with the overall performance of the bacterial communities in colonizing skin models in a manner that yields human-relevant outcomes, particularly with respect to bacterial functionality and maintenance of diversity, without disrupting tissue integrity or inducing cytotoxicity.

The BAP.10 community, with the highest initial species and phyla diversity, reached the highest overall score across all assessed endpoints. The decisive feature was its superior ability to mimic the functional profile of the human skin microbiome. Skin colonization by the BA.8 community, which had the second-highest initial species diversity, resulted in outcomes similar to those of the BAP.10 community. However, this community did not sufficiently mimic the functional profile of the human skin microbiome and, therefore, was ranked second. These were followed by the BP.6 and AP.6 communities, which produced comparable outcomes, albeit with the AP.6 community inducing slightly higher AK levels. In contrast, the P.2, followed by the B.4 and A.4 communities, received the lowest overall scores, primarily due to bacterial counts that did not reflect in vivo conditions, combined with a substantial loss of bacterial diversity, insufficient functional capacity, impaired skin integrity, and cytotoxic effects in the co-culture skin model. These findings demonstrate the importance of bacterial diversity and sufficient functional coverage for human-relevant skin colonization in vitro.

## 4. Discussion

Full-thickness in vitro skin models are well established and are increasingly employed as alternatives to animal models for a broad range of applications [76]. Incorporation of the microbiome faces obstacles in mimicking the complexity of host–microbiome interactions and longevity. Most studies are limited to several hours or up to five days or inoculation of skin with individual taxa, or neglect to assess bacterial viability [12,24,77,78]. In this context, we characterized the interaction of seven bacterial communities of varying diversity—which comprised the phyla Bacillota (B), Actinomycetota (A), and Pseudomonadota (P)—and human skin in vitro. To disentangle the effects of bacterial colonization per se and the role of bacterial diversity in microbiota–skin interactions, we analyzed both, the skin tissue and the microbiota, in detail.

Despite all individual species being added in equal ratios, their relative abundances from 16S sequencing were uneven already in the inocula. These discrepancies may be explained by 16S rRNA gene amplicon sequencing biases. Multiple gene copies, sequencing primers, clustering methods, reference database, and analysis parameters can lead to sequencing biases such as unequal amplification, which affect bacterial composition ratios [79,80,81]. The low abundance of *Staphylococcus* spp. in both the inocula and day 7 samples may also be attributed to impeded cell lysis, likely due to the robust staphylococcal cell wall characterized by a highly cross-linked muropeptide fraction and long oligomeric chains [82]. Furthermore, we cannot exclude that certain species were underrepresented in the community analyses due to the harvesting approach. In addition to rigorous and repeated DPBS washing, no additional mechanistic disruptions were applied, to preserve tissue integrity for subsequent skin analyses. Hence, strong adherence or biofilm formation may contribute to a reduced recovery. To ensure comparability, all skin models were processed using the same standardized protocol, while accepting this limitation.

While the bacterial counts of the three- and two-phyla communities BAP.10, BA.8, BP.6, and AP.6 reached in vivo-like numbers [32,83] and demonstrated superior α-diversity, with over 90% (BAP.10) at the end of cultivation, the single-phylum communities A.4, B.4, and P.2 either harbored no viable bacteria or outnumbered the in vivo-like counts. For example, *Pseudomonas* sp. was the dominant species in the P.2 co-cultures. These findings align with a previous study, in which skin models inoculated with *Pseudomonas oleovorans* (Pseudomonadota) reached up to 10^11^ CFUs/skin model [17]. Bacteria present in the phylum Pseudomonadota degrade a wide range of xenobiotics [84] and have extensive lipid metabolism [85,86], also mirrored by their increased representation of pathways related to lipid metabolism and xenobiotic biodegradation, including steroid hormone biosynthesis and cytochrome P450-associated drug metabolism in the functional profiling analysis. These abilities may give *Pseudomonas* spp. a competitive advantage in nutrient-poor environments and thus, account for the high cell counts and subsequent destruction of skin tissues [87]. Increased abundance of bacteria, including *Pseudomonas* spp., have been documented in skin infections and wounds [88,89,90], and chronic diseases, such as atopic dermatitis [91], together with a reduction in bacterial diversity [5,92,93,94,95].

In contrast to the P.2 community, the B.4 community comprising the *Staphylococcus* species *S. capitis, S. warneri, S. condimenti,* and *S. epidermidis* did not colonize the skin models. This could be related to their limited functional potential, manifested in the fewest functional KEGG categories resulting in the most distinct functional profile from both the BAP.10 community and the SMGC. The lack of complete KEGG metabolic modules suggests limited metabolic capacity and further metabolic dependence [96], which is in line with the inability of the B.4 community to successfully colonize the skin models. Such dependence could pose significant challenges, particularly in nutrient-poor environments and when metabolic cooperation with other bacteria is absent. Given the nutrient-poor environment of the skin, metabolite exchange plays a key role in microbial colonization, survival, and community stability [97,98]. Another reason for the inability of the B.4 community to independently colonize the skin models could be the higher sensitivity to host-derived antimicrobial peptide (AMP) expression [99] or intra-genus competition. *Staphylococci* are among the most frequent inhabitants of human skin [2] and compete with other species under nutrient-limited conditions, particularly through growth-inhibiting bacteriocins [5,100]. Such competition is pronounced among closely related species that share metabolic niches [101]. Effective colonization by *Staphylococcus* spp. has been reported when *Staphylococcus* spp. is accompanied by other genera [102]. This mirrors our findings, as the Bacillota together with the Pseudomonadota (BP.6) exceeded the functional potential of the two respective phyla alone. When comparing the initial relative abundance of *Staphylococcus* spp. with harvested samples, an increase was observed in all *Staphylococcus*-containing viable co-cultures. For instance, together with Actinomycetota species, the functional profiling resembled those of the skin microbiota reference and showed similar lipid, carbohydrate, and amino acid metabolism. These functions are essential for skin barrier function and pathogen protection [8,21,96,103,104] and our findings suggest that the Actinomycetota species provide these metabolic functions relevant for growth of *Staphylococcus* spp. and are generally critical for similarity to the human skin microbiome, as represented by the skin microbiota reference. Our findings highlight the importance of multidirectional signaling and cross-feeding to maintain a diverse microbiota [95,105]. This multidirectional interaction on the one hand may prevent the predominance of individual species that otherwise outcompete others and disrupt the community balance and, on the other hand, may be key for successful colonization for metabolically dependent microbes [97,98].

Transcriptomic analysis served as a descriptive tool to visualize overall gene expression changes potentially relevant for microbiota–skin interaction. Colonization with the BAP.10 community induced metabolic pathways involved in ‘regulation of cell cycle processes’, ‘cilium organization’, and ‘glycoprotein synthesis’, thereby suggesting a beneficial role of BAP.10 colonization in maintaining skin homeostasis and barrier formation, given that glycoprotein synthesis is crucial for the skin’s barrier function and defense against pathogens [106,107,108]. This is further indicated by the preservation of structural integrity, a slight enhancement in epidermal thickness, and elevated levels of E-cadherin and Cytokeratin 10, particularly in the BAP.10 and BA.8 co-cultures. E-cadherin is critical for the epidermal barrier as it regulates tight junctions [74]. The differentiation marker Cytokeratin 10 has been reported to enhance the structural integrity of the *stratum corneum*, and its absence was associated with impaired cutaneous permeability and reduced *stratum corneum* hydration [109,110,111]. Cytokeratin 10 and E-cadherin are frequently used as markers in panels to assess epidermal structural maturation and cell–cell adhesion within the epidermal layers [112]. Both markers have previously been shown to be modulated by the presence of human skin commensals, indicating their potential relevance for evaluating microbiota-induced alterations of epidermal barrier function [113]. The latter and other studies indicate a beneficial role of commensal bacterial communities for the skin’s barrier function and pathogen protection in mice [21,114] and humans [17,19,24,115]. Compared to individual species, a diverse microbial community more effectively influenced expression of genes associated with key cell cycle processes, increased epidermal thickness, and enhanced cell proliferation in a full-thickness skin model [24], which underlines the importance of a diverse microbial community in maintaining balanced and physiologically relevant skin colonization in vitro.

While differential gene expression in tissues colonized by the BAP.10, BA.8, BP.6, and to a lesser extent, AP.6 communities was relatively low compared to aseptic tissues, colonization with the A.4 community exhibited significant induction of genes. Younis et al. colonized surgically prepared breast tissue for 24 h with commensal, opportunistic pathogenic, or pathogenic bacteria [116]. While commensal bacteria had minimal effect on the transcriptional profile, pathogenic bacteria induced a pronounced change in host gene expression, particularly associated with apoptosis and reduced expression of differentiation markers. Our findings suggest that this is applicable not only to individual pathogens but also to low diversity. Such conditions can precipitate excessive bacterial proliferation that culminates in an infectious state and a compromised skin barrier [5,95]. The low-diversity A.4 and P.2 communities compromised the skin integrity, accompanied by significant cellular damage and cytotoxicity. Although the A.4 community was composed of abundant skin commensals, the harvested communities were still among the least diverse communities, dominated by *Kocuria* sp., and loss of individual species or dominance by others are associated with bacterial growth in low-niche in vitro systems [117,118]. Another hypothesis is that generalist species within the A.4 community transition to opportunists when conditions are favorable, thereby outcompeting other species [87]. Transcriptome analysis of colonized skin tissue with the A.4 community suggested a disrupted cellular homeostasis, visible in substantially different gene expression profiles. There, pathways involved in ‘apoptotic signaling’ or ‘modification of kinase activity’ were induced, which are concomitant during oxidative stress or DNA damage [119] or after infection [116]. Upregulation of the metabolic pathways ‘neutrophil migration’ and ‘autophagy’ imply an inflammatory response and cellular recycling due to tissue infection or injury [120]. Altogether, these findings suggest a pathological condition in skin tissues colonized by the A.4 community, which is in line with studies correlating reduced bacterial diversity and skin diseases [5,92,95].

Even with these advancements, it is important to recognize certain limitations of the study. As such, the limited number of replicates has to be carefully considered when drawing broad and substantiated conclusions from the data and the transcriptomic findings of this study should be regarded as indicative trends that require validation and confirmation through qPCR in subsequent studies to validate the relevance of our findings in host–microbiome crosstalk. We are also aware that the most diverse defined community, BAP.10, lacks common aerobic and anaerobic skin commensals. The inclusion of other species or strains as well as altering the proportions of bacteria in the community may influence some of our findings, such as the colonization itself, the composition of and functions provided by the established communities, and with that, the related parameters in the tissue. The functional analysis provided within the study is based on whole-genome sequences and reflects the functional potential of the microbial communities. However, they neither capture the actual activity of specific metabolic pathways nor the enzymatic or metabolic output generated in the skin co-cultures. Future studies should address this limitation by including metabolome or metaproteome analyses to identify the pathways and metabolites that are utilized under specific co-culture conditions and that mediate the interaction between the skin microbiota and the underlying tissues [121,122] for cause–effect analysis. Commensal skin models could be further improved by incorporating special niches, such as hair follicles and sweat glands, to expand the nutrient pool and enable co-culture with other commensal (facultative) anaerobic bacteria and specialists such as *Cutibacterium acnes* or *Corynebacterium* spp. This is likely to influence bacterial composition, and thus, skin homeostasis. In addition, given that the skin microbiome interacts closely with host immune cells to protect against pathogens and maintain skin homeostasis [123], the incorporation of immune cells may enhance the model’s physiological relevance. These additional suggestions underscore the necessity to develop both biologically relevant microbiota–skin co-cultures and advanced in vitro skin models to more accurately mimic human skin in vivo.

In this context, our work represents a critical step towards mimicking the complexity of host–microbes and microbe–microbe interactions, in a defined longevity colonized model system of human skin.

## 5. Conclusions

Overall, this study allows a comprehensive understanding of individual contributions between the bacterial communities and their suitability for skin colonization. We address a critical gap in modeling host–microbe interactions in healthy human skin by systematically characterizing a co-culture system of human skin bacteria with full-thickness skin models that maintains a diverse and viable bacterial community over seven days in vitro. Our findings highlight the importance of bacterial diversity and the presence of different genera to achieve human-relevant functionality and sustain greater diversity alongside physiological bacterial counts during co-cultivation with human skin cells, thereby preventing cytotoxicity and inflammation. This was observed in the most diverse co-culture BAP.10. Hence, the BAP.10 community may serve as a defined community for future host–microbiota studies. Our findings provide valuable insights for and should be taken into account when developing defined commensal communities for skin co-cultures and to study host–microbiome interactions.

## Figures and Tables

**Table 1:** Composition of bacterial communities for skin inoculation.

Species	Community Name						
	BAP.10	BA.8	BP.6	AP.6	B.4	A.4	P.2
*Staphylococcus capitis*							
*Staphylococcus condimenti*							
*Staphylococcus epidermidis*							
*Staphylococcus warneri*							
*Kocuria rhizophila*							
*Micrococcus luteus* strain 1							
*Micrococcus luteus* strain 2							
*Brevibacterium frigoritolerans*							
*Acinetobacter radioresistens*							
*Pseudomonas fulva*							
Inoculum [bacterial cells/strain]	1 × 10^4^	1.25 × 10^4^	1.67 × 10^4^	1.67 × 10^4^	2.5 × 10^4^	2.5 × 10^4^	5 × 10^4^
Inoculum [total bacterial cells/skin model]	1 × 10^5^						

The background color indicates the presence of species in the respective community.

## Data Availability

Raw and processed transcriptome data files have been deposited in the Gene Expression Omnibus (GEO) data repository under accession number GSE283830. Raw 16S rRNA gene amplicon sequencing data files are available in the European Nucleotide Archive (ENA) under project number PRJEB90404. Additional datasets can be obtained from the corresponding author upon reasonable request.

## Supplementary Materials

The following supporting information can be downloaded at https://www.mdpi.com/article/10.3390/microorganisms14081722/s1: Figure S1: α- and β-diversities of the bacterial communities used for inoculation and from the co-cultures; Figure S2: Whole genome-based analyses of bacterial functional potential per species; Figure S3: Distance matrix comparing the functional potential of all seven defined communities and microbiomes from the SMGC based on Jaccard distance using annotated KO terms; Figure S4: Heatmap visualization of bacteria-relevant KEGG modules arranged in KEGG classes and subclasses and clustered for community similarity; Figure S5: Dermal transcriptomic profiles of colonized and aseptic tissue; Figure S6: Epidermal transcriptomic profiles of selected markers in colonized tissues; Figure S7: HE staining of colonized and aseptic models; Figure S8: Tissue integrity of colonized and aseptic models; Table S1: Characteristics of the bacterial skin isolates; Table S2: Similarity of epidermal transcriptomic profiles on day 7; Table S3: Relative abundances.

## Abbreviations

The following abbreviations are used in this manuscript:

A	Actinomycetota
AAs	Auxiliary Activities
AK	Adenylate Kinase
B	Bacillota
BSA	Bovine Serum Albumin
CAZy	Carbohydrate-active Enzymes
CBM	Carbohydrate-binding Modules
CEs	Carbohydrate Esterases
CFU	Colony Forming Unit
CI	Confidence Interval
ECVAM	European Centre for the Validation of Alternative Methods
FGF	Fibroblast Growth Factor
GHs	Glycoside Hydrolases
GO	Gene Ontology
GSEA	Gene Set Enrichment Analysis
GTs	Glycosyltransferases
IMNGS2	Integrated Microbial Next-generation sequencing 2
KEGG	Kyoto Encyclopedia of Genes and Genomes
KO	KEGG ontology
LinDA	Linear Models for Differential Abundance Analysis
MAG	Metagenome-assembled Genome
NES	Normalized Enrichment Score
OECD	Organisation for Economic Co-operation and Development
P	Pseudomonadota
PCA	Principal Component Analysis
PCoA	Principal Coordinate Analysis
PCR	Polymerase Chain Reaction
PL	Polysaccharide Lyases
RIN	RNA-integrity
RMA	Robust Multiarray Average
SMGC	Skin Microbial Genome Collection
SOTU	Species-like Operational Taxonomic Unit
t_D_	Doubling Time
TdT	Terminal Deoxynucleotidyl Transferase
TIC	Taxonomy Informed Clustering
