# Supplementary material for: Elucidating the Impact of Bacterial Composition and Diversity on Skin Homeostasis Using a Human 3D In Vitro Skin Model
Source: Microorganisms. 2026 Aug 5;14(8):1722. doi: 10.3390/microorganisms14081722 (PMC13515475; doi:10.3390/microorganisms14081722)
Supplement: Supplementary file 1 [file microorganisms-14-01722-s001.zip › Supplemental information_1.pdf]

microorganisms

MDPI

Supplementary Materials

Table S1. Characteristics of the bacterial skin isolates.

Species with Weihenstephan Strain (WS) collection number [30]	Colony color	Gram type	Oxygen requirement	Doubling time [h]	OD_{600} max
k_Bacteria;p_**Bacillota**;c_Bacilli;o_Caryophanales;f_Staphylococcaceae; g_*Staphylococcus;s_capitis* WS 5788	beige	+	facultative anaerobe	0.69 ± 0.15	10.33 ± 1.45
k_Bacteria;p_**Bacillota**;c_Bacilli;o_Caryophanales;f_Staphylococcaceae; g_*Staphylococcus;s_condimenti* WS 5790	white	+	facultative anaerobe	0.59 ± 0.12	10.76 ± 0.81
k_Bacteria;p_**Bacillota**;c_Bacilli;o_Bacillales;f_Staphylococcaceae; g_*Staphylococcus;s_epidermidis* WS 5794	beige	+	facultative anaerobe	0.77 ± 0.07	17.03 ± 1.91
k_Bacteria;p_**Bacillota**;c_Bacilli;o_Caryophanales;f_Staphylococcaceae; g_*Staphylococcus;s_warneri* WS 5789	beige	+	facultative anaerobe	0.75 ± 0.09	2.17 ± 0.71
k_Bacteria;p_**Actinomycetota**;c_Actinomycetes;o_Micrococcales; f_Micrococcaceae;g_*Kocuria;s_rhizophila* WS 5787	yellow	+	obligate aerobe	1.34 ± 0.04	13.98 ± 5.55
k_Bacteria;p_**Actinomycetota**;c_Actinomycetes;o_Micrococcales; f_Micrococcaceae;*g_Micrococcus;s_luteus* WS 5784	yellow	+	obligate aerobe	1.34 ± 0.05	21.57 ± 6.07
k_Bacteria;p_**Actinomycetota**;c_Actinomycetes;o_Micrococcales; f_Micrococcaceae;*g_Micrococcus;s_luteus* WS 5785	white	+	obligate aerobe	1.29 ± 0.47	10.83 ± 1.17
k_Bacteria;p_**Actinomycetota**;c_Actinomycetes;o_Micrococcales; f_Brevibacteriaceae;g_*Brevibacterium;s_frigoritolerans* [1]Bacteria;**Bacillota**;Bacilli;Caryophanales;Bacillaceae;*Peribacillus simplex* WS 5786	beige	+	aerobe	0.54 ± 0.05	15.01 ± 6.72
k_Bacteria;p_**Pseudomonadota**;c_Gammaproteobacteria;o_Pseudomonadales; f_Moraxellaceae;g_*Acinetobacter;s_radioresistens* WS 5792	beige	–	obligate aerobe	1.02 ± 0.22	6.72 ± 0.39
k_Bacteria;p_**Pseudomonadota**;c_Gammaproteobacteria;o_Pseudomonadales; f_Pseudomonadaceae;g_*Pseudomonas;s_fulva* WS 5791	yellow	–	obligate aerobe	0.59 ± 0.09	4.31 ± 0.21

[1] The bacterium *Brevibacterium frigoritolerans* was previously assigned to Actinomycetota, however, it was reclassified in 2022 as *Peribacillus frigoritolerans*, belonging to Bacillota. Given that the experiments were conducted prior to this reclassification, we decided to retain this strain in Actinomycetota for the sake of this study.

https://doi.org/10.3390/microorganisms14081722

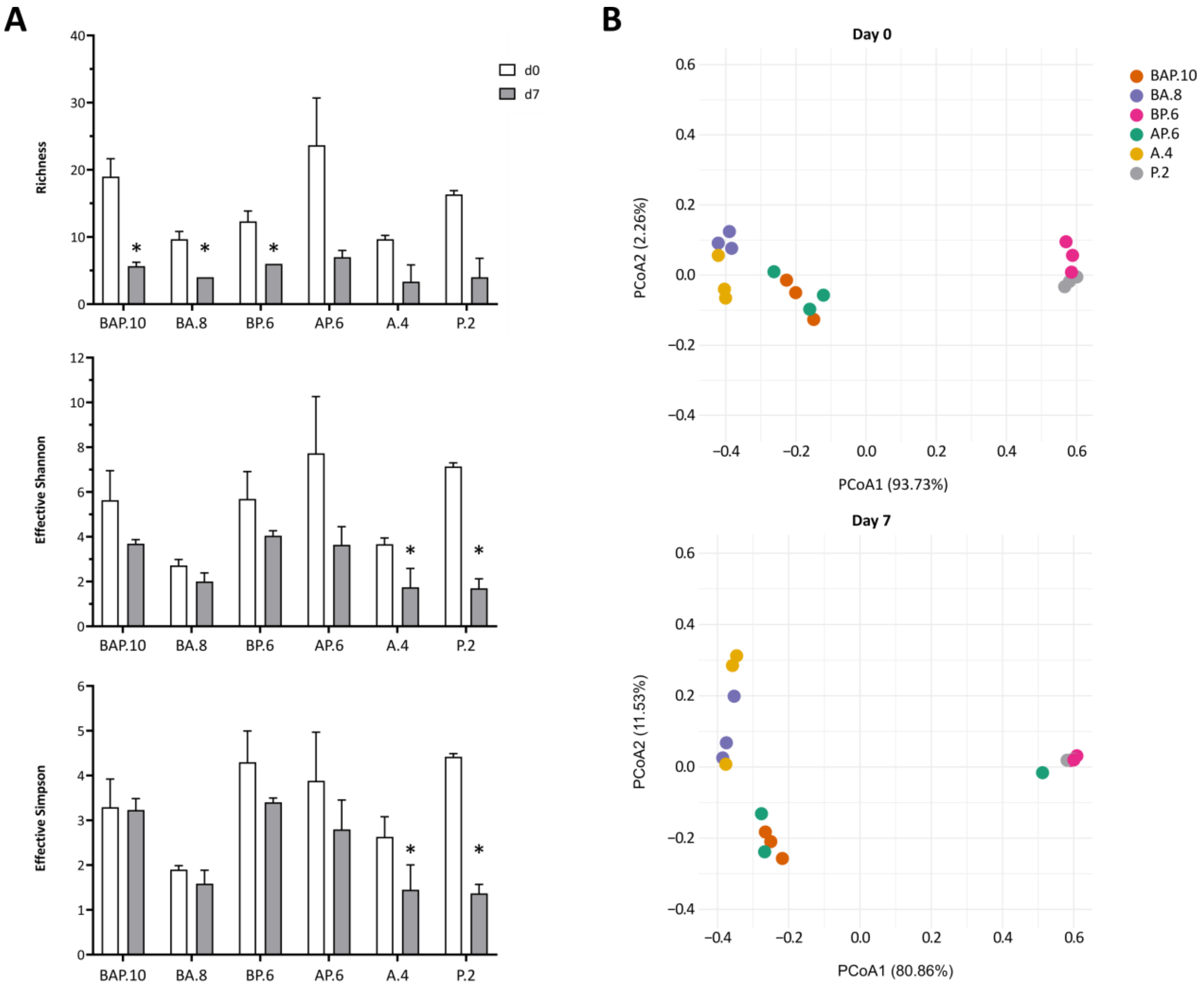

Figure S1. α- and β-diversities of the bacterial communities used for inoculation and from the co-cultures. All bacterial communities were subjected to 16S rRNA gene amplicon sequencing of the V1-V3 on the day of inoculation and at the end of co-culture on day 7. (A) α-Diversity of the respective bacterial communities on day 0 compared to day 7, presented as richness, Shannon and Simpson effective values, and (B) β-diversities visualized in PCoA plots. Significance levels were determined using multiple paired two-tailed t-test with *$p < 0.05$. Abbreviations: B = Bacillota; A = Actinomycetota; P = Pseudomonadota.

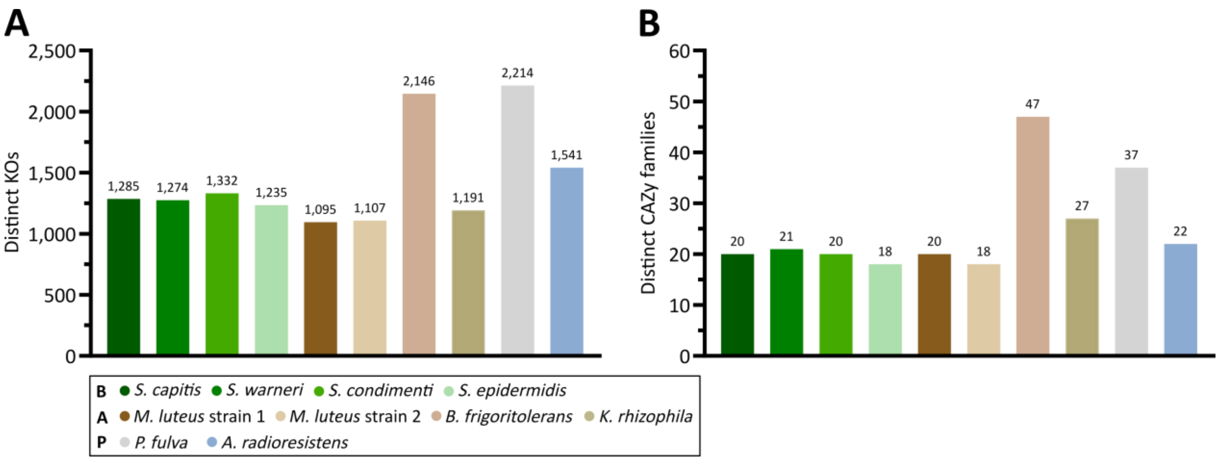

Figure S 2. Whole genome-based analyses of bacterial functional potential per species. (A) Number of annotated KEGG orthology terms. (B) Number of CAZyme families within the individual whole genomes.

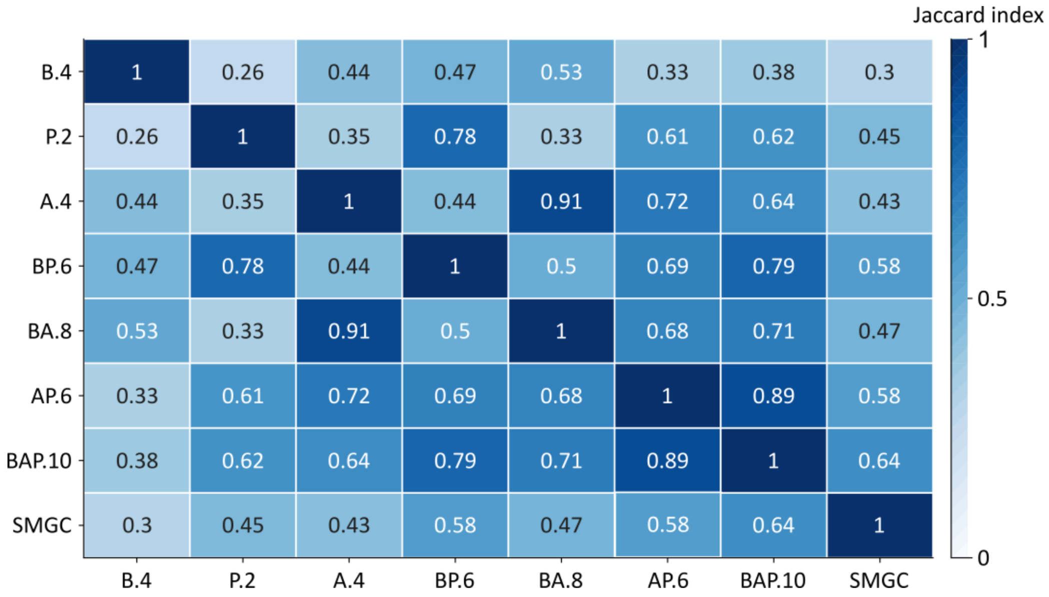

Figure S 3. Distance matrix comparing the functional potential of all seven defined communities and microbiomes from the SMGC based on Jaccard distance using annotated KO terms.

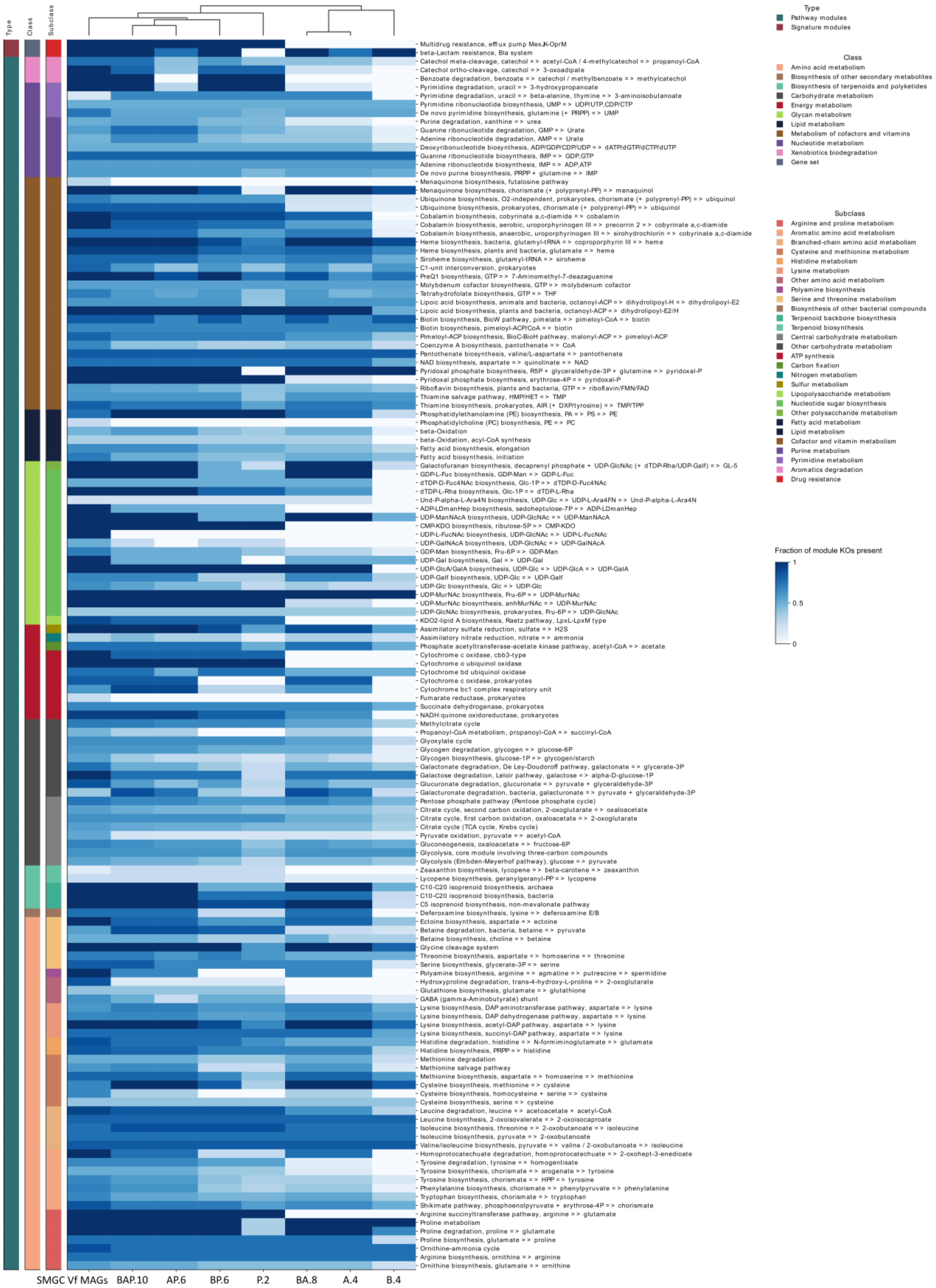

Figure S 4. Heatmap visualization of bacteria-relevant KEGG modules arranged in KEGG classes and subclasses and clustered for community similarity. Color indicates the fraction of the respective KEGG modules representing module completeness.

Table S2. Similarity of epidermal transcriptomic profiles on day 7.[1]

#genes	Comparison of co-cultures	p-value	p-value.mixed
**21194**	A.4 vs all others	0.2820211	0.04230215
	A.4 vs aseptic models	0.1181776	0.16564900
	A.4 vs BAP.10	0.2000218	0.01267867
	A.4 vs BA.8	0.3317533	0.12241430
	A.4 vs BP.6	0.6033771	0.10850340
	A.4 vs AP.6	0.7692124	0.02638573

[1] Global epidermal transcriptome-wide differences between the A.4 co-cultures and the other co-cultures were assessed using the global rotation test (fry) implemented in the limma package. The mixed-direction p-value (p.mixed) was used to test for overall differences in expression profiles, irrespective of the direction of individual gene changes.

https://doi.org/10.3390/microorganisms14081722

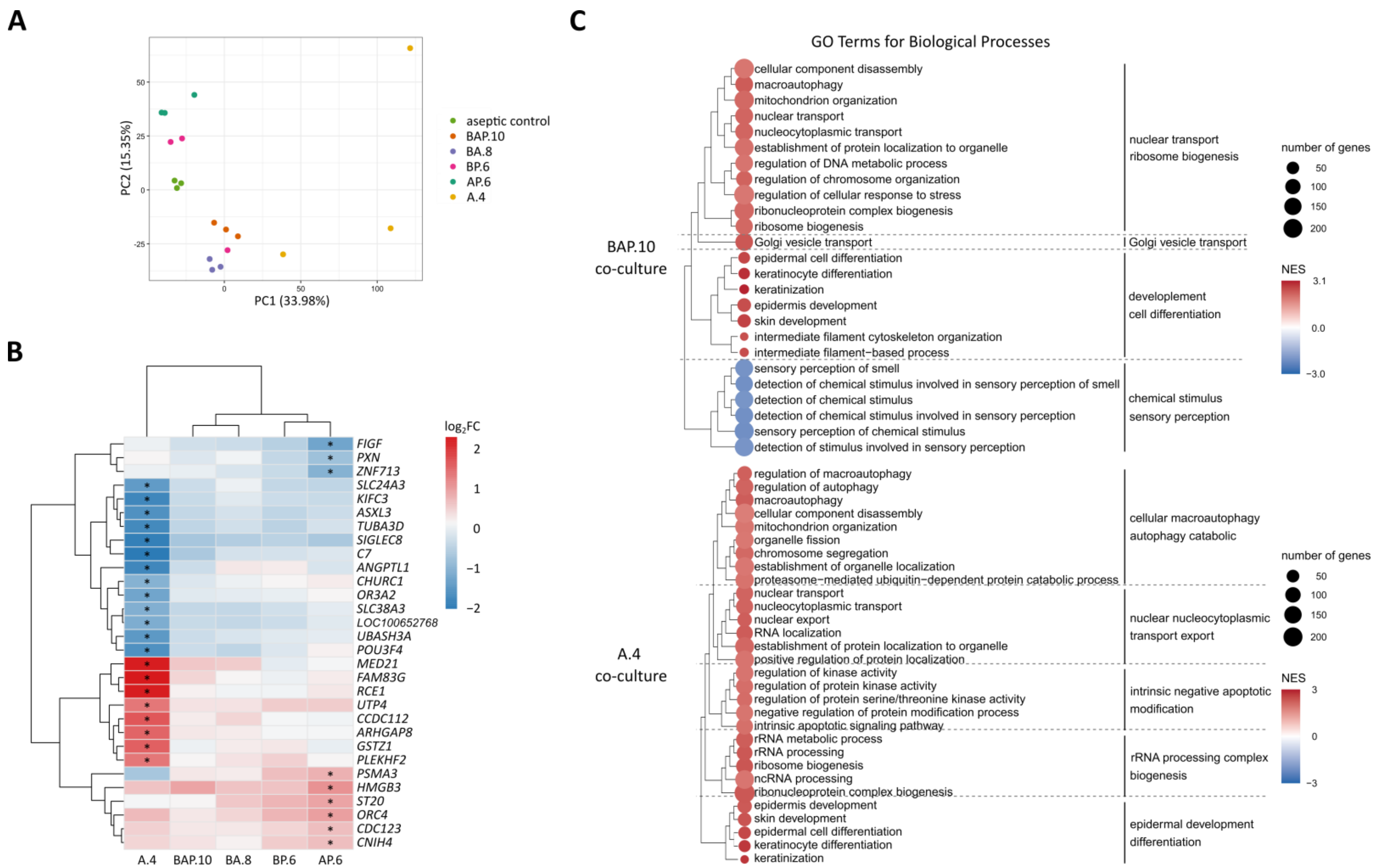

Figure S5. Dermal transcriptomic profiles of colonized and aseptic tissue. (A) PCA-plot for showing the similarity in dermal transcription profiles analyzed on day 7. (B) Heatmap with cluster dendrograms for both rows and columns for log_2FC of all significantly differentially expressed genes in the dermis of colonized tissues compared to aseptic tissue. (C) Treeplots of GSEA identifying enriched GO terms for biological processes in the dermis of BAP.10- and A.4-colonized tissue normalized to aseptic tissue on day 7 (n = 3). The dot size represents the number of genes per functional group and the color code refers to NES. Significance levels are presented as adjusted p-value with *adj. $p < 0.05$. Abbreviations: B = Bacillota; A = Actinomycetota; P = Pseudomonadota.

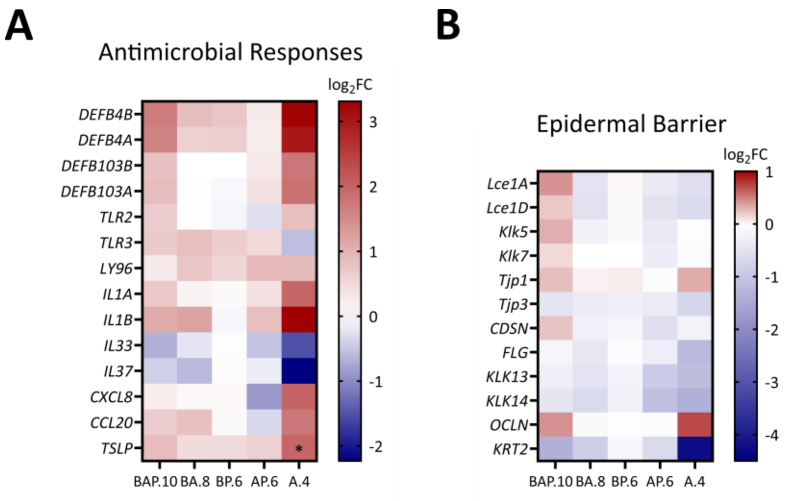

Figure S6. Epidermal transcriptomic profiles of selected markers in colonized tissues. Epidermal log_2FC of selected markers involved in (A) antimicrobial responses and (B) epidermal barrier on day 7 are displayed. Significance levels are presented as adjusted p-value with *adj. $p < 0.05$. Abbreviations: B = Bacillota; A = Actinomycetota; P = Pseudomonadota.

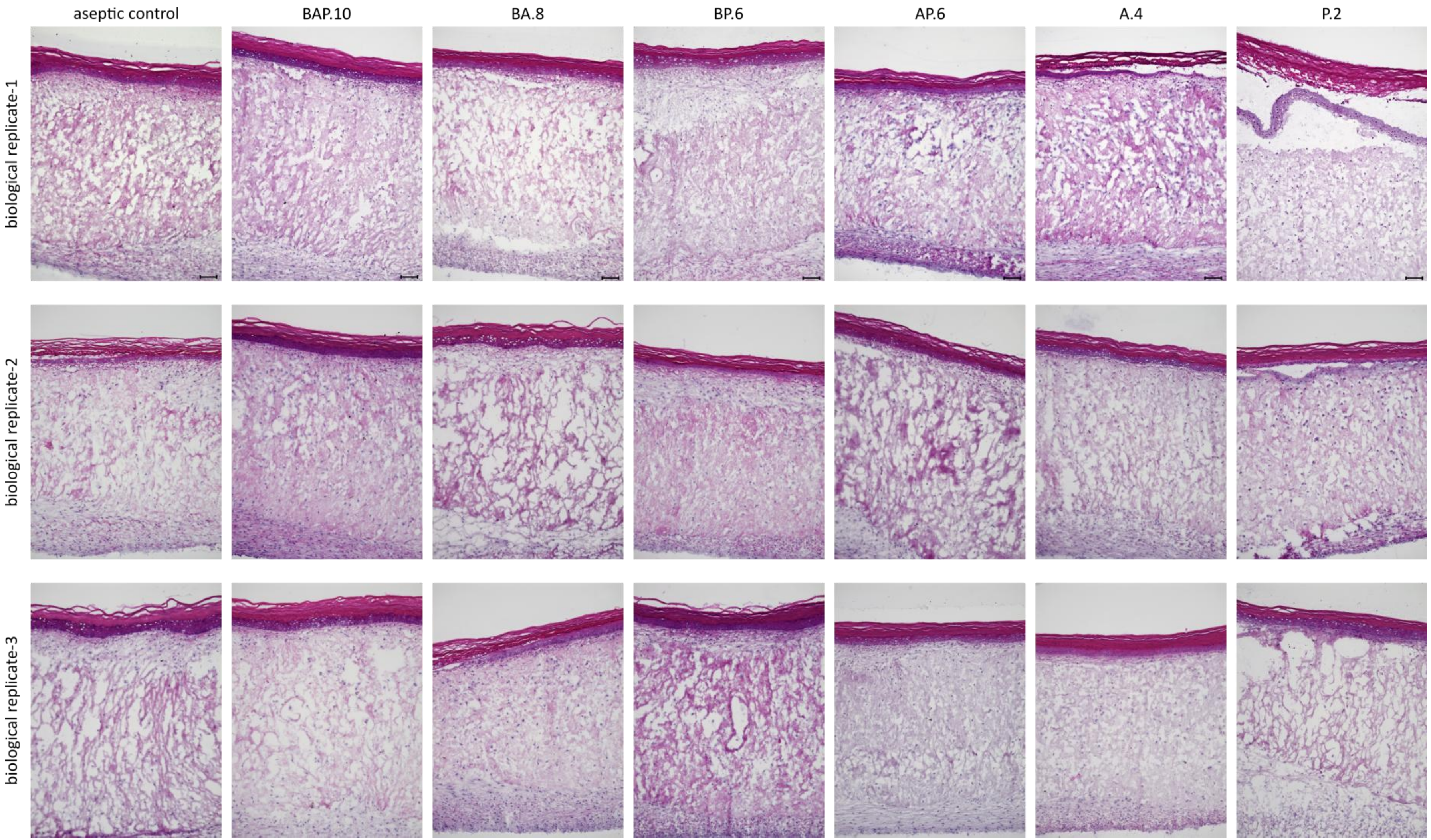

Figure S7. HE staining of colonized and aseptic models. Hematoxylin and eosin (HE) staining images from inoculated and aseptic models of three biological replicates on day 7 of cultivation, with scale bars indicating 100 µm. Abbreviations: B = Bacillota; A = Actinomycetota; P = Pseudomonadota.

microorganisms

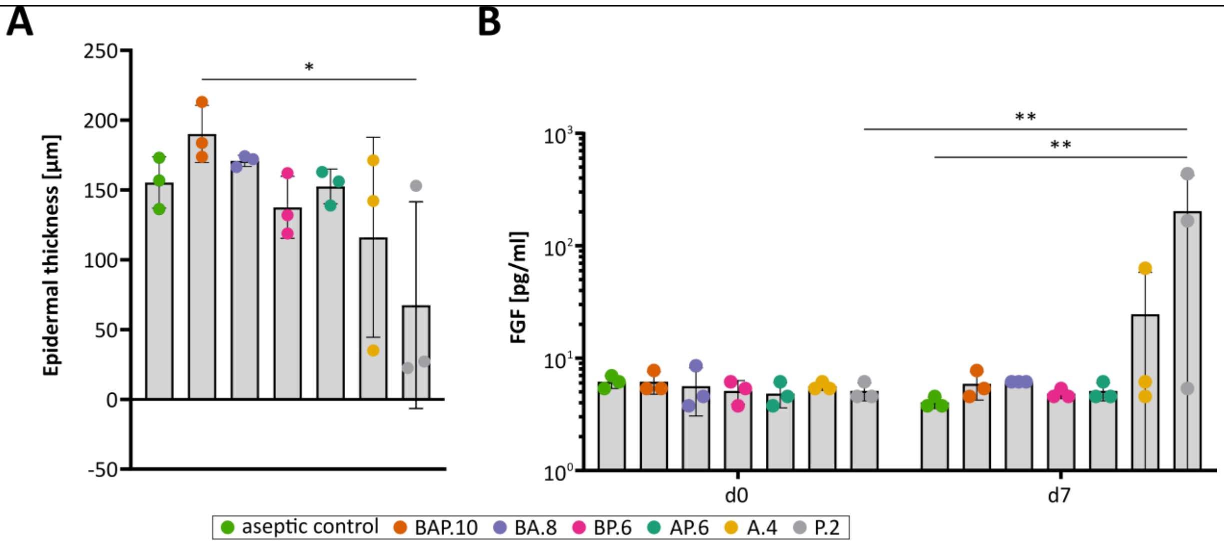

Figure S8. Tissue integrity of colonized and aseptic models. (A) Epidermal thickness was measured across ten different regions of the HE staining of three biological replicates per group. Differences between the control and the inoculated co-culture were analyzed using one-way ANOVA, followed by Tukey's multiple comparisons test. Adjusted p-values are reported (*adj. $p < 0.05$). (B) Bar plots for quantification of FGF released into the supernatant of colonized and aseptic control models on day 0 and day 7 based on ELISA measurements of three biological replicates. Data are shown as mean ± SD. Co-cultures were compared to aseptic control and changes estimated over time using two-way ANOVA with Tukey's multiple comparisons test. Adjusted p-values are reported (**adj. $p < 0.01$). Abbreviations: B = Bacillota; A = Actinomycetota; P = Pseudomonadota.

Disclaimer/Publisher's Note: The statements, opinions and data contained in all publications are solely those of the individual author(s) and contributor(s) and not of MDPI and/or the editor(s). MDPI and/or the editor(s) disclaim responsibility for any injury to people or property resulting from any ideas, methods, instructions or products referred to in the content.

https://doi.org/10.3390/microorganisms14081722
